# Isoginkgetin protects against degeneration of ALS motor neurons via regulating the GSK-3β–TFEB signaling axis

**DOI:** 10.1016/j.phrs.2026.108172

**Published:** 2026-05

**Authors:** Ang Li, Xianglu Xiao, Guopan Liu, Krinos Li, Yixia Ling, Shenglong Deng, Chunzuan Xu, Shu-Qin Cao, Jing Wen, Guang Lu, Guang Yang, Evandro F. Fang, Dajiang Qin, Huanxing Su

**Affiliations:** a State Key Laboratory of Mechanism and Quality of Chinese Medicine, Institute of Chinese Medical Sciences, University of Macau, Macao, China; b Bioengineering Department and Imperial-X, Imperial College London, London W12 7SL, United Kingdom; c Guangdong Engineering Technology Research Center of Biological Targeting Diagnosis, Therapy and Rehabilitation, The Fifth Affiliated Hospital, Guangzhou Medical University, No.621 Gangwan Road, Guangzhou, China; d Centre for Regenerative Medicine and Health, Hong Kong Institute of Science & Innovation, Chinese Academy of Sciences, Hong Kong, China; e Department of Clinical Molecular Biology, University of Oslo and Akershus University Hospital, Lørenskog 1478, Norway; f Department of Physiology, Zhongshan School of Medicine, Sun Yat-sen University, Guangzhou, China; g National Heart and Lung Institute, Imperial College London, London SW7 2AZ, United Kingdom; h Cardiovascular Research Centre, Royal Brompton Hospital, London SW3 6NP, United Kingdom; i School of Biomedical Engineering & Imaging Sciences, King's College London, London WC2R 2LS, United Kingdom; j The Norwegian Centre on Healthy Ageing (NO-Age) and the Norwegian National anti-Alzheimer’s Disease (NO-AD) Networks, Oslo, Norway

**Keywords:** Amyotrophic lateral sclerosis, GSK-3β–TFEB axis, Isoginkgetin, Lysosome, Artificial intelligence

## Abstract

Lysosomal dysfunction is a core pathological driver of neurodegenerative diseases such as amyotrophic lateral sclerosis (ALS). Transcription factor EB (TFEB) serves as a master regulator of lysosomal biogenesis, and its pharmacological activation represents a strategy to restore lysosomal function in disease and aging. Here, using a series of artificial intelligence-powered computational virtual screening workflows, we have identified isoginkgetin (ISO), a small-molecule compound, as a potent TFEB activator that promotes mechanistic target of rapamycin complex 1 (mTORC1)–independent TFEB nuclear translocation to enhance lysosomal biogenesis and function. Mechanistically, ISO functions as an ATP-competitive inhibitor that binds to the key Lys85 residue within the ATP-binding pocket of glycogen synthase kinase 3β (GSK-3β), thereby regulating the GSK-3β–TFEB signaling axis to activate TFEB nuclear translocation. Functionally, ISO improves lysosomal function and protects motor neurons differentiated from induced pluripotent stem cells derived from patients with ALS from degeneration. Collectively, these results support the hypothesis that lysosomal dysfunction is a druggable target for ALS.

## Graphical Abstract

**Figure:**
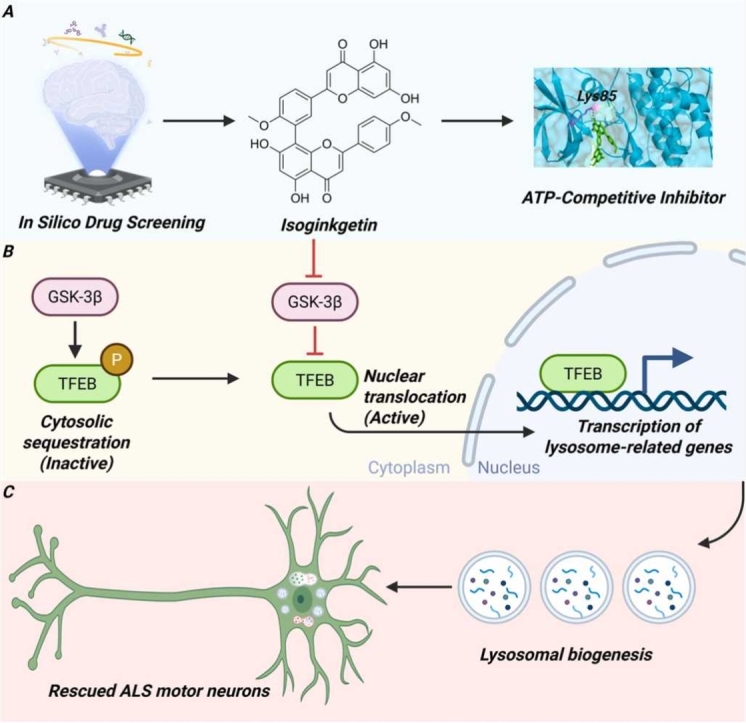

## 1 Introduction

Amyotrophic lateral sclerosis (ALS), also known as Lou Gehrig’s disease, is a rare, progressive, and fatal neurodegenerative disease primarily affecting motor neurons in the brain and spinal cord, causing progressive muscle weakness and atrophy ultimately leading to death from respiratory failure [1], [2]. The lysosome is a key organelle responsible for intracellular degradation in eukaryotic cells; it plays a vital role in maintaining cellular homeostasis and preserving cellular viability [3]. Lysosomal dysfunction has been implicated in ALS. Several ALS-associated genes, such as *C9orf72*, *TARDBP*, and *TBK1*, are involved in regulating lysosomal function; mutations in these genes impair lysosome function, leading to motor neuron degeneration [4], [5]. Consequently, restoring or enhancing lysosomal function has emerged as a promising druggable therapeutic strategy for ALS.

A diverse array of therapeutic strategies has been developed to improve lysosomal dysfunction [6], [7], [8]. One of these strategies is the targeted activation of transcription factor EB (TFEB), a master regulator of lysosomal biogenesis, representing a potential therapeutic strategy for lysosomal dysfunction [9]. TFEB activity is closely related to its subcellular localization; it is either retained in the cytoplasm (inactive state) or translocated into the nucleus (active state) after dephosphorylation[10]. This localization switch is precisely regulated by a panel of kinases mediating the phosphorylation of specific amino acid residues on TFEB. Among these regulatory kinases, mechanistic target of rapamycin complex 1 (mTORC1) acts as the most crucial upstream inhibitory kinase of TFEB [11]. Numerous mTORC1-dependent TFEB activators, including Rapamycin (Sirolimus), have been developed and applied in multiple clinical trials for neurodegenerative diseases [12], [13]. Considering the crucial physiological role of mTOR, which is an essential regulator governing nutrient sensing, cellular metabolism, growth, proliferation, and immune homeostasis, therapeutic strategies relying on mTOR inhibition to modulate TFEB nuclear translocation carry inherent risks of disrupting these fundamental cellular processes, potentially leading to some adverse effects [14]. This highlights the need to develop new drugs that can regulate TFEB nuclear translocation in an mTOR-independent manner.

Recent advances in artificial intelligence (AI) have been successfully applied in accelerating the identification of novel compound structures involved in autophagy and mitophagy [15], [16], [17], [18]. This study was conducted to develop an AI-powered virtual screening workflow integrating ligand- and structure-based approaches to identify mTOR-independent TFEB activators. The complexity of neurodegenerative pathways was addressed by applying AI-assisted ligand-based screening to a library of more than one million compounds and then performing large-scale cross-docking of the top-ranked candidates against hundreds of autophagy-related proteins. This comprehensive and scalable workflow represents one of the large-scale cross-docking applications within the autophagy field. It has enabled systematic identification of functionally relevant small molecules at an unprecedented depth and breadth. Using this workflow, we identified a novel mTORC1-independent TFEB agonist Isoginkgetin (ISO). This TFEB agonist promoted TFEB nuclear translocation by directly binding to glycogen synthase kinase 3 beta (GSK-3β) and inhibiting its activity, thereby mitigating ALS-related pathologies driven by *TARDBP* (the gene encoding the TDP-43 protein) and *SOD1* mutations in motor neurons.

## 2 Results

### 2.1 Virtual screening identifies potential mTOR-independent TFEB nuclear translocation agonists

**Fig. 1:**
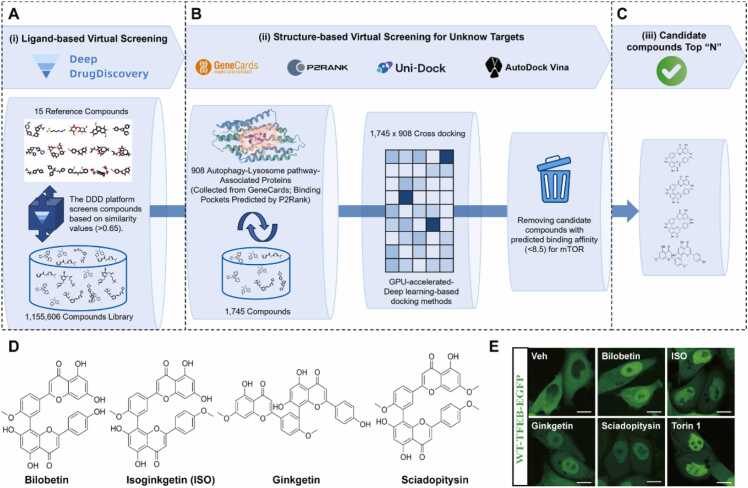
GPU-accelerated virtual screening pipeline for identifying novel mTOR-independent TFEB nuclear translocation agonists. The workflow for the virtual screening process: (A) DDD platform employed AI-driven LBVS technology to screen a large commercial compound library (*n* = 1155,606). Based on structural similarity scores (>0.65), it identified 1745 candidate compounds with structural similarity to known mTOR-independent TFEB nuclear translocation agonists. (B) For the structure-based virtual screening, the workflow generated a cross-docking matrix comprising 1745 compounds and 908 proteins related to the autophagy–lysosome pathway. Of these, compounds displaying minimal affinity to mTOR but strong binding across multiple proteins related to the autophagy–lysosome pathway were prioritized. (C) Candidate molecules were ranked by their average docking scores, and the top 20 were selected. (D) Chemical structures of four AI-identified compounds (bilobetin, ginkgetin, ISO, and sciadopitysin) belonging to the 3′-8''-biflavone family, derived from *Ginkgo biloba*, are shown. (E) Live-cell imaging showing that all four compounds (10 μM, 12 h) induced the TFEB nuclear translocation in TFEB-EGFP HeLa cells, using Torin 1 (250 nM, 2 h) as the positive control. Scale bars, 25 μm.

To identify potential mTOR-independent TFEB nuclear translocation agonists, we performed AI-driven ligand-based virtual screening (LBVS) using the Deep Drug Discovery (DDD) platform (Fig. 1A), which was built by the combinational molecular representation method as we reported previously [15]. Here, a total of 15 known mTOR-independent TFEB nuclear translocation agonists were used as reference structures (Table S1). The DDD platform integrated multi-level molecular representations [one-dimensional (1D), two-dimensional (2D), and three-dimensional (3D) descriptors] via an attention-based learning framework. For 15 known reference structures, it ranked each compound from a large commercial compound library (*n* = 1155,606) by their structural similarity scores (Fig. 1A). We identified 1745 candidates (similarity values greater than 0.65, a threshold provided by the platform), all of which shared structural similarity with existing (known) mTOR-independent TFEB nuclear translocation agonists (Fig. 1B). Then, we used P2Rank to predict the binding pockets of 908 proteins associated with the autophagy–lysosome pathway. This protein list was constructed based on autophagy-related proteins from GeneCards, excluding those involved in the composition of the mTOR complex (Table S2) [19]. Graphics processing unit (GPU)-accelerated Uni-Dock engine was employed to generate a cross-docking matrix involving 1745 compounds and 908 target proteins [20]. We used large-scale structure-based virtual screening to calculate the docking scores of the compounds with all proteins and ranked the candidate compounds based on the docking scores (Figs. 1B and 1C). We also excluded candidate compounds with potential affinity for mTOR (Fig. 1B). Detailed information on the final top 20 in silico–selected candidate compounds is documented in Table S3. We were interested to find four natural compounds: Bilobetin, Ginkgetin, ISO, and Sciadopitysin (Fig. 1D), belonging to the 3′-8''-biflavone family derived from *Ginkgo biloba* among these top 20 compounds [21]. Biological experiments using wild type (WT)-TFEB-EGFP HeLa cells showed that all these four compounds significantly promoted TFEB nuclear translocation (Fig. 1E).

### 2.2 ISO promotes mTORC1-independent TFEB nuclear translocation

**Fig. 2:**
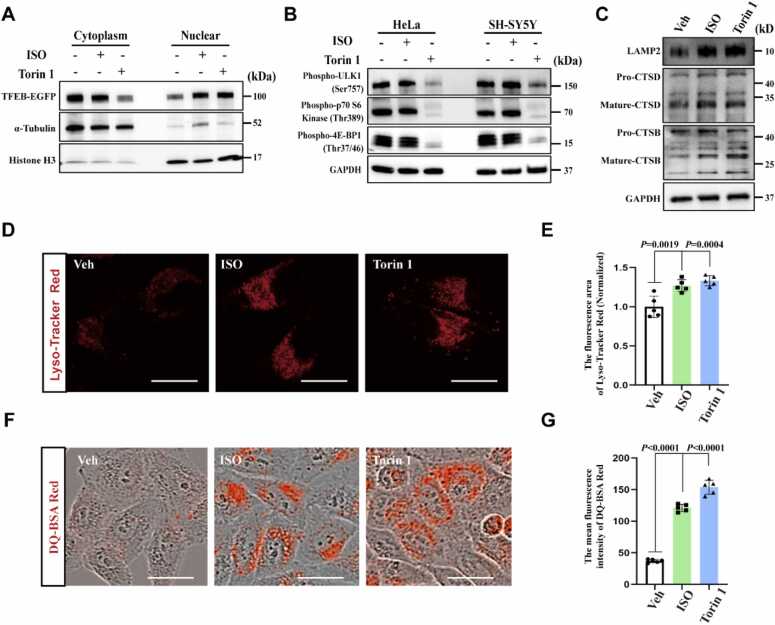
ISO promotes TFEB nuclear translocation and enhances lysosomal biogenesis and function. (A) Nucleus–cytoplasm fractionation assay showing that ISO (10 μM, 12 h) treatment promoted TFEB nuclear translocation in TFEB-EGFP HeLa cells, using Torin 1 (250 nM, 6 h) as the positive control. (B) Western blotting results showing that ISO (10 μM, 12 h) did not inhibit the activity of mTORC1 in HeLa and SH-SY5Y cells, using Torin 1 (250 nM, 6 h) as the positive control. (C) Western blotting results showing that ISO (10 μM, 12 h) increased the expression of lysosome-related proteins, using Torin 1 (250 nM, 6 h) as the positive control. (D and E) Live-cell imaging results showing that ISO (10 μM, 12 h) increased the number of functional lysosomes indicated by LysoTracker Red dye, using Torin 1 (250 nM, 6 h) as the positive control; *n* = 5, five biological replicates. Scale bars, 25 μm. (F and G) Live-cell imaging results showing that ISO (10 μM, 12 h) increased the lysosomal activity as indicated by the DQ-BSA-Red dye staining, using Torin 1 (250 nM, 6 h) as the positive control; *n* = 5, five biological replicates. Scale bars, 25 μm. Data were presented as the mean ± standard deviation. One-way ANOVA was followed by Dunnett's multiple comparison test.

Our recent report identified ISO as a safe and effective lead compound with therapeutic potential in ALS [22], prompting us to explore the underlying mechanisms by which ISO activated TFEB among the four AI-selected compounds from the 3′-8′′-biflavone family. Live-cell imaging revealed that ISO promoted the nuclear translocation of TFEB in (WT)-TFEB-EGFP HeLa cells in a dose-response manner (Fig. S1A). Immunoblot analysis confirmed that ISO increased the protein expression level of TFEB in the cell nucleus (Fig. 2A) without altering phosphorylation of the three downstream substrates of mTORC1 including phospho-ULK1 (Ser757), phospho-p70 S6 Kinase (Thr389), and phospho-4E-BP1 (Thr37/46), suggesting that ISO activated TFEB independent of mTORC1 activity (Fig. 2B). TFEB is phosphorylated at residues S122 and S211 under mTORC1 regulation [23]. We then mutated the serine (S) residues at positions 122 and 211 of the TFEB protein to aspartic acid (D) to mimic phosphorylation modification. Notably, introduction of the S122D/S211D mutation failed to abrogate ISO-induced TFEB nuclear translocation, thereby further validating that ISO induces TFEB nuclear translocation via an mTORC1-independent mechanism (Fig. S1B–S1C). Then, immunoblot analysis showed a significant increase in the expression levels of the lysosomal membrane protein marker lysosomal-associated membrane protein 2 (LAMP2) and the mature forms of two lysosomal hydrolases, mature-cathepsin B (mature-CTSB) and mature-cathepsin D (mature-CTSD), after ISO treatment (Fig. 2C). Two probes were used to further verify whether ISO enhanced lysosomal biogenesis and function: LysoTracker Red dye staining to determine the total number of acidic, functionally intact lysosomes [24], and DQ-BSA-Red dye staining to assay overall lysosomal hydrolytic activity [25]. We found that ISO increased the number of functional lysosomes, as indicated by the increase in the fluorescent area of LysoTracker Red dye (Figs. 2D and 2E), and enhanced the hydrolase activity of lysosomes, as reflected by the increased fluorescent intensity of the DQ-BSA-Red dye (Figs. 2F and 2G). Similar results were also confirmed in the SH-SY5Y cells (Fig. S1D–S1F). Under this condition as previously reported [26], ISO did not impair ubiquitin–proteasome system (UPS) function (Fig. S1G). All these lines of evidence substantiate that ISO promotes mTORC1-independent TFEB nuclear translocation and enhances lysosomal biogenesis and function.

### 2.3 ISO inhibits GSK-3β activity both *in vitro* and *in vivo*

**Fig. 3:**
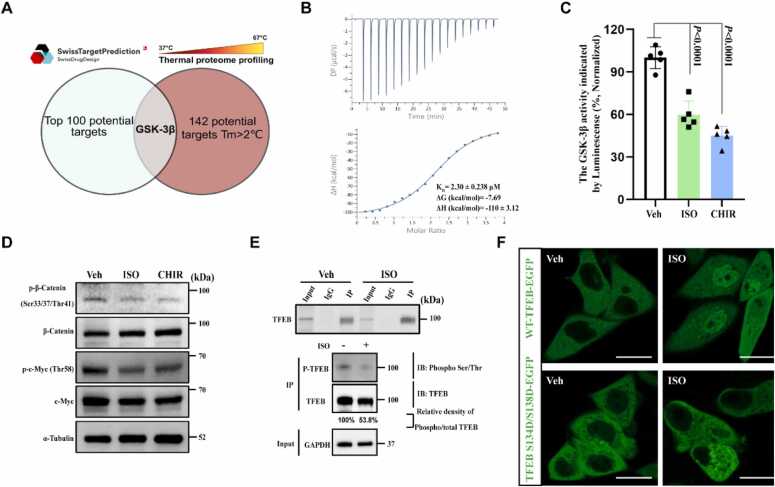
ISO binds directly to GSK-3β and inhibits its activity. (A) Online molecular-target prediction tool SwissTargetPrediction, combined with TPP results, predicted GSK-3β as a potential binding target of ISO. (B) ISO binding to GSK-3β protein was determined by isothermal titration calorimetry. (C) ISO (10 μM) inhibited GSK-3β activity *in vitro*, using CHIR-99021 (1 μM; CHIR) as the positive control; *n* = 5, five biological replicates. (D) Activity of downstream proteins of GSK-3β in cells was detected by Western blotting, revealing that ISO (10 μM, 12 h) inhibited GSK-3β activity *in vivo*, using CHIR-99021 (1 μM, 4 h) as the positive control. (E) Immunoprecipitation result showed that the phosphorylation level of TFEB protein decreased after treating TFEB-EGFP HeLa cells with ISO (10 μM, 12 h), indicating that ISO inhibited GSK-3β activity. (F) Transfection of the TFEB S134D/S138D mutant plasmid into HeLa cells mimicked the sequential phosphorylation of TFEB by GSK-3β. The ability of ISO to induce TFEB nuclear translocation was blocked. Scale bars, 25 μm. Data were presented as the mean ± standard deviation. One-way ANOVA was followed by Dunnett's multiple comparison test.

We then explored how ISO activated TFEB. To identify the molecular targets through which ISO promotes TFEB nuclear translocation, we performed thermal proteome profiling (TPP), and used the SwissTargetPrediction online tool to predict candidate molecular targets as previously described [27] (Fig. S2A). SwissTargetPrediction provided the top 100 potential binding targets (Table S4), whereas TPP identified 142 proteins with a melting temperature (*T*_m_) shift greater than 2°C (Table S5). We integrated the results from both methods, identifying a shared target protein, GSK-3β (Fig. 3A). GSK-3β maintains TFEB in an inactive state by directly phosphorylating serine residues at positions 134 and 138 of TFEB; however, inhibiting its activity can induce mTORC1-independent nuclear translocation of TFEB [28]. Isothermal titration calorimetry (ITC, *K*_D_ = 2.30 ± 0.238 μM, Fig. 3B) and cellular thermal shift assay (CETSA, Fig. S2B and S2C) showed that ISO could directly bind to the GSK-3β protein both *in vitro* and *in vivo*. *In vitro* kinase activity assay demonstrated that ISO inhibited GSK-3β kinase activity, as indicated by decreased luminescence intensity (Fig. 3C; IC_50_ = 16.97 μM, Fig. S2D). Western blot analysis revealed that ISO treatment inhibited the expression of GSK-3β downstream substrates, including Phospho-β-Catenin (Ser33/37Thr41) and Phospho-c-Myc (Thr58) (Fig. 3D) and reduced the phosphorylation level of TFEB *in vivo* (Fig. 3E). Notably, the construction and transfection of the S134D/S138D TFEB mutant to mimic phosphorylation at these residues [29], blocked the ability of ISO to induce TFEB nuclear translocation (Fig. 3F). Several pathways such as PI3K/Akt, MAPK, Ca²⁺/PKC, and Wnt/β-catenin are involved in regulating GSK-3β activities [28], [30], [31], [32]. We performed co-treatment of ISO with specific inhibitors for the upstream kinases (PI3K/Akt inhibitor LY294002, MAPK/p90RSK inhibitor BI-D1870, pan-PKC inhibitor Go6976, and Wnt/β-catenin pathway inhibitor XAV939) to rule out the possibility that ISO inhibited GSK-3β kinase activity by regulating upstream kinases rather than through direct binding of GSK-3β. We found that ISO still promoted TFEB nuclear translocation (Fig. S2E). Thus, we concluded that ISO is directly bound to GSK-3β kinase to inhibit its activity.

### 2.4 ISO competitively binds to the GSK-3β kinase ATP pocket

**Fig. 4:**
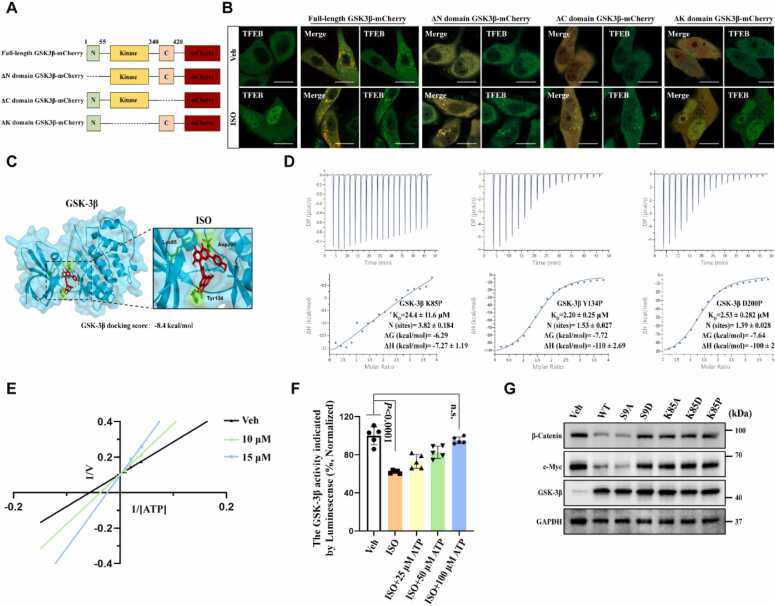
ISO an ATP-competitive inhibitor of GSK-3β. (A) Schematic diagram of the construction of GSK-3β truncated plasmids. (B) Live-cell imaging results showed that the overexpression of full-length GSK-3β blocked ISO-induced TFEB nuclear translocation, but when the kinase domain of GSK-3β was truncated, this blocking effect disappeared. Scale bars, 50 μm. (C) Molecular docking model of ISO binding to GSK-3β protein showed that ISO formed hydrogen bonds with the Lys85, Tyr134, and Asp200 amino acids in the GSK-3β protein. (D) ITC showed that, among the three different mutants of GSK-3β protein (K85P, Y134P, and D200P), the GSK-3β K85P mutant protein significantly impacted the direct binding between ISO and GSK-3β protein. *N* ≈ 1 suggests that the protein might have only one ligand-binding site. (E) Lineweaver–Burk plot derived from the kinetic data of GSK-3β kinase activity, measured under treatment with 10 and 15 μM ISO, revealed that ISO was an ATP-competitive inhibitor of GSK-3β; *n* = 3, three biological replicates. (F) *In vitro* GSK-3β kinase activity assays showed that supplementation with high concentrations of ATP (100 μM) reversed the inhibitory effect of ISO on GSK-3β; *n* = 5, five biological replicates. (G) Western blotting results showed that the degradation of GSK-3β downstream substrates was not promoted after transfecting HeLa cells with three GSK-3β Lys85 mutant plasmids (100 ng; including GSK-3β K85A, GSK-3β K85D, and GSK-3β K85P), indicating that these mutants lacked kinase activity. Data were presented as the mean ± standard deviation. One-way ANOVA was followed by Dunnett's multiple comparison test.

We constructed various truncated forms of GSK-3β to identify the binding site of ISO on GSK-3β (Fig. 4A). Western blot analysis revealed successful construction and expression of all truncated forms of GSK-3β in HeLa cells (Fig. S3A). We found that the overexpression of wild-type GSK-3β protein blocked ISO-induced TFEB nuclear translocation; however, this inhibitory effect was abolished when the kinase domain of GSK-3β was truncated (Fig. 4B). The docking analysis with the CB-Dock2 server (used for protein–ligand blind docking [33]) showed that ISO formed hydrogen bonds with Lys85 (K85), Tyr134 (Y134), and Asp200 (D200) amino acid residues located in the kinase domain of the GSK-3β protein (Fig. 4C). All these three binding sites were located within the ATP-binding pocket of GSK-3β [34]. We obtained protein mutants of three potential binding sites through protein purification: GSK-3β K85P, GSK-3β Y134P, and GSK-3β D200P (Fig. S3B). ITC results showed that the affinity of ISO for GSK-3β K85P protein was significantly reduced, as evidenced by a more than tenfold increase in the *K*_D_ value (Fig. 4D), suggesting K85 of GSK-3β as a potential binding site for ISO. Kinetic experiments were performed by varying the concentrations of both ATP (20, 50, and 100 μM) and ISO (10 and 15 μM). Lineweaver–Burk plotting of the data suggested that ISO served as an ATP-competitive inhibitor for GSK-3β, as for the maximal velocity (*V*_max_) of the reaction was found to be almost constant across various ISO concentrations (all the straight lines intersected on the 1/*v* axis, Fig. 4E), which was consistent with the kinetic profiles of GSK-3β ATP-competitive inhibitors reported previously [35]. This finding was confirmed by an *in vitro* kinase assay, showing that supplementation with high concentrations of ATP (100 μM) abolished the ability of ISO to inhibit GSK-3β activity (Fig. 4F). Mutation of the K85 residue in GSK-3β resulted in GSK-3β inactivation [36], [37], [38]. We overexpressed three K85 mutants (K85A, K85D, and K85P) in HeLa cells, along with two well-characterized mutants S9A (active) and S9D (inactive) [39], to verify that K85 was a critical binding site located within the ATP pocket of GSK-3β and closely related to GSK-3β activity. β-Catenin and c-Myc have been reported to undergo destabilization upon phosphorylation by active GSK-3β [40], [41]. Western blot analysis showed that, unlike the active wild type-GSK-3β or GSK-3β S9A protein, none of the three K85 mutants could promote the degradation of β-Catenin and c-Myc (Fig. 4G). Moreover, unlike the wild type-GSK-3β protein, the three K85 mutants also could not block ISO-induced TFEB nuclear translocation (Fig. S3C and S3D). Thus, we concluded that ISO, as an ATP-competitive inhibitor of GSK-3β, inhibited its activity through directly binding to the K85 amino acid residue of GSK-3β.

### 2.5 Lysosomal dysfunction in ALS motor neurons

**Fig. 5:**
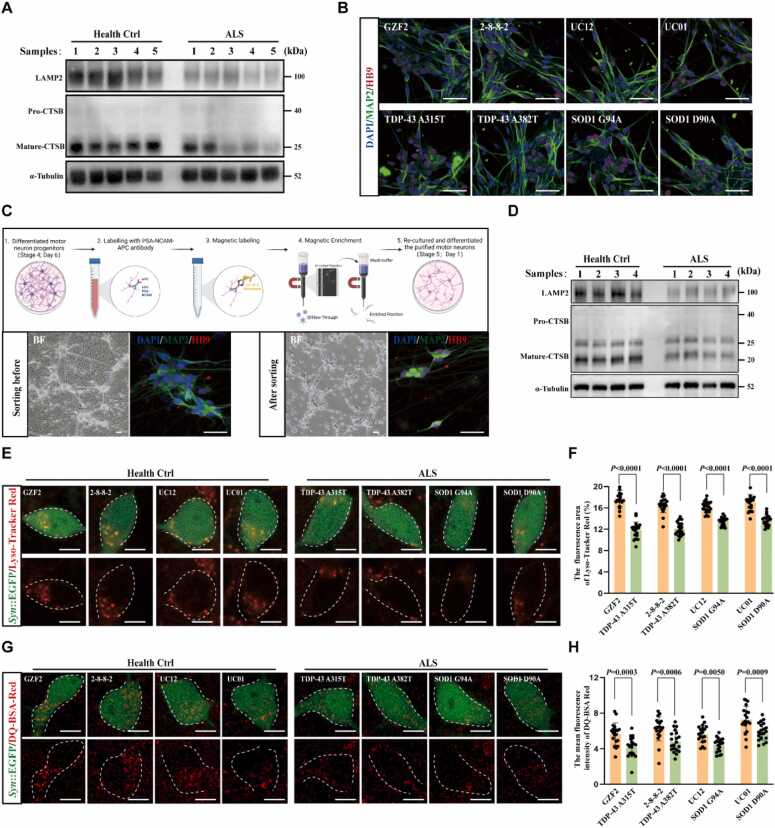
Lysosomal dysfunction in samples from patients with ALS and iPSC-derived motor neurons. (A) Western blotting was performed to detect the expression of lysosome-related proteins in the brain tissues (Middle frontal gyrus) from patients with ALS and healthy controls. The results revealed lysosomal dysfunction in the samples from patients with ALS. (B) Immunostaining of motor neurons derived from eight iPSC lines on day 10 in stage 5 demonstrating the differentiation of MAP2^+^/HB9^+^ motor neurons. Cellular nuclei were counterstained with DAPI. Scale bars, 100 μm. (C) Schematic diagram of magnetic microbead sorting for motor neurons. As observed in the immunofluorescence and bright-field images, MAP2^+^/HB9^+^ motor neurons displayed significant enrichment. Scale bars, 100 μm. (D) Western blotting analysis was performed to detect the expression of lysosome-related proteins in purified day-28 ALS motor neurons and healthy controls. The results indicated lysosomal dysfunction in ALS motor neurons. Health ctrl 1: GZF2; Health ctrl 2: 2–8–8–2; Health ctrl 3: UC12; Health ctrl 4: UC01; ALS 1: TDP-43 A315T; ALS 2: TDP-43 A382T; ALS 3: SOD1 G94A; ALS 4: SOD1 D90A. (E) Representative live-cell imaging images of 28-day-old motor neurons labeled with *Syn*::EGFP and treated with LysoTracker Red dye. Scale bars, 10 μm. (F) Quantitative results of (E) showed a significant reduction in the number of functional lysosomes in ALS motor neurons; *n* = 20 motor neurons for each group. (G) Representative live-cell imaging images of 28-day-old motor neurons labeled with *Syn*::EGFP and treated with DQ-BSA-Red dye. Scale bars, 10 μm. (H) Quantitative results of (G) showed a significant decrease in the lysosomal activity in ALS motor neurons; *n* = 20 motor neurons for each group. Data were presented as the mean ± standard deviation. Two-way ANOVA was followed by Tukey’s multiple comparison test.

We then investigated lysosome function in the brain tissue sample of patients with ALS and sex-matched healthy controls from the Netherlands Brain Bank (Table S6). Immunoblot analysis revealed a reduction in the expression of LAMP2 and mature-CTSB in patients with ALS, suggesting the presence of lysosomal dysfunction (Fig. 5A). ALS iPSC-derived motor neurons and controls were used to further examine lysosomal dysfunction in ALS. The iPSCs derived from patients with ALS included two commercially available cell lines: *TARDBP A382T* mutant iPSCs and *SOD1 D90A* mutant iPSCs (WiCell Research). To ensure that the ALS-specific phenotypes are not due to inherent variability between cell lines, we generated isogenic controls by introducing the heterozygous TDP-43 A315T and SOD1 G94A mutations into the healthy GZF2-iPSC line and UC12-iPSC line, respectively (Fig. S4A). Four healthy control iPSC lines, UC12-iPSC, GZF2-iPSC, UC01-iPSC, and 2–8–8–2-iPSC were maintained in our lab [22], [42], [43], [44]. All iPSC cell lines were differentiated into MAP2^+^ /HB9^+^ motor neurons (Fig. 5B). We performed magnetic sorting, a technique that purifies target motor neuron progenitors by leveraging the specific binding between PSA-NCAM on progenitors’ surface and magnetic microbead-conjugated antibodies, to enrich motor neurons in iPSC-derived cultures (Fig. 5C) [45]. Immunofluorescence results showed that this sorting led to an enrichment of more than 85% MAP2^+^/HB9^+^ motor neurons (Fig. S4B and S4C). Based on this, we performed Western blot analysis. We found that the expression of LAMP2 and mature-CTSB was also reduced in ALS motor neurons on day 28 of maturation (Fig. 5D). The human *synapsin 1* (*hSYN1*) gene reporter system has been reported to label choline acetyltransferase (ChAT)-positive motor neurons *in vivo*[46]. We found that the *Syn*::EGFP reporter system could label viable MAP2^+^/HB9^+^ motor neurons after 21 days of motor neuron maturation (Fig. S4D). Using LysoTracker Red and DQ-BSA-Red dye staining, we found that *Syn*::EGFP-labeled ALS motor neurons on day 28 exhibited a significant reduction in functional lysosome number and impaired lysosomal activity compared with their healthy controls (Fig. 5E–H). These results collectively demonstrated lysosomal dysfunction in ALS motor neurons.

### 2.6 ISO improves lysosome function and antagonizes ALS-like pathology in iPSC-derived motor neurons

**Fig. 6:**
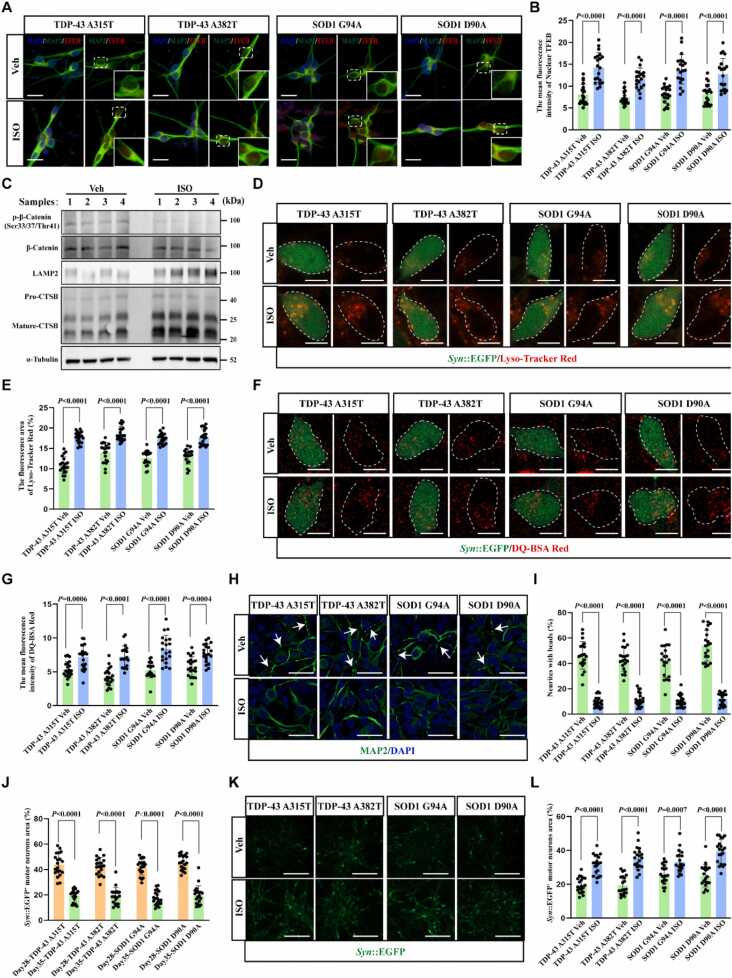
ISO improves lysosomal function in ALS motor neurons via the GSK-3β–TFEB signaling axis. (A) Representative immunofluorescence images for detecting TFEB nuclear translocation induced by ISO (750 nM, 24 h) in 28-day-old ALS motor neurons. Scale bars, 20 μm. (B) Quantitative results of (A) showed that ISO induced TFEB nuclear translocation in four types of ALS motor neurons; *n* = 20 motor neurons for each group. (C) Western blotting was performed to detect the expression of lysosome-related proteins in purified day-28 ALS motor neurons treated with ISO (750 nM, 24 h). The results indicated that ISO inhibited GSK-3β activity and increased the expression of lysosome-related proteins. Sample 1: TDP-43 A315T; Sample 2: TDP-43 A382T; Sample 3: SOD1 G94A; and Sample 4: SOD1 D90A. (D) Representative live-cell imaging images of 28-day-old motor neurons labeled with *Syn*::EGFP and treated with LysoTracker Red dye after ISO (750 nM, 24 h) treatment. Scale bars, 10 μm. (E) Quantitative results of (D) showed that ISO increased the number of functional lysosomes in ALS motor neurons; *n* = 20 motor neurons for each group. (F) Representative live-cell imaging images of 28-day-old motor neurons labeled with *Syn*::EGFP and treated with DQ-BSA-Red dye after ISO (750 nM, 24 h) treatment. Scale bars, 10 μm. (G) Quantitative results of (F) showed that ISO enhanced the lysosomal activity in ALS motor neurons; *n* = 20 motor neurons for each group. (H) Representative images of neurite swelling in ALS motor neurons after 7-day ISO (750 nM) treatment detected by immunofluorescence (white arrow). Scale bars, 50 μm. (I) Quantitative results of (H) showed that ISO reduced the proportion of neurite bead-like swelling in ALS motor neurons; *n* = 20 images for each group. (J) Quantification results of the area of *Syn*::EGFP-labeled ALS motor neurons from day 28 to day 35 showed a gradual loss of EGFP signals, which was attributed to the progressive death of ALS motor neurons; *n* = 20 images for each group. (K) Representative images of *Syn*::EGFP-labeled ALS motor neurons, captured via live-cell imaging after 7-day ISO (750 nM, from day 28 to day 35) treatment. Scale bars, 200 μm. (L) Quantitative results of (K) showed that ISO increased the number of surviving *Syn*:: EGFP^+^ ALS motor neurons; *n* = 20 images for each group. Data were presented as the mean ± standard deviation. Two-way ANOVA was followed by Tukey’s multiple comparison test.

ISO was used for treating four types of ALS motor neurons. Initial dose–response control experiments revealed no cellular toxicity in iPSC-derived motor neurons in the presence of 750 nM ISO for 7 days (Fig. S5A). The nuclear TFEB expression level in the four ISO-treated ALS motor neurons was higher than that in untreated controls (Figs. 6A and 6B). Western blot analysis showed that ISO-treated motor neurons exhibited reduced expression of Phospho-β-Catenin (Ser33/37Thr41) and a significant increase in LAMP2 and mature-CTSB expression (Fig. 6C), indicating that ISO activated the GSK-3β–TFEB axis to promote lysosome biogenesis in ALS motor neurons. LysoTracker Red and DQ-BSA-Red dye staining further confirmed that both the number of functional lysosomes and the activity of lysosomal hydrolases were significantly elevated in ISO-treated ALS motor neurons (Fig. 6D–6 G). Moreover, ISO significantly reduced the proportion of neurites with bead-like swellings in ALS motor neurons and decreased the release of lactate dehydrogenase (LDH) after 7 days of treatment (Figs. 6 H, 6I, and S5C). ISO treatment significantly increased the number of surviving *Syn*::EGFP-labeled motor neurons at day 35 of maturation, compared with untreated controls (Fig. 6J–6 L). Meanwhile, GSK-3β overexpression in ALS motor neurons blocked the ISO-induced upregulation of LAMP2 and mature-CTSB expression, thereby attenuating the protective effect of ISO on ALS motor neurons, as reflected by LDH release (Fig. S5B and S5C). In summary, our findings showed that ISO enhanced lysosomal biogenesis and function by promoting TFEB nuclear translocation and antagonized ALS-like pathology in ALS motor neurons.

## 3 Discussion

The integration of AI into virtual screening has markedly improved the efficiency and precision of early-stage drug discovery with success [15]. In this study, the AI-driven LBVS approach enabled rapid prioritization of compounds with high structural and physicochemical similarities to known TFEB activators from an ultra-large chemical library. The DDD platform leveraged attention-based molecular representations to effectively capture subtle molecular features underlying TFEB potential activation. This data-driven filtering substantially reduced the search space and guided subsequent structure-based analyses toward biologically meaningful candidates. These approaches helped efficiently identify four compounds belonging to the 3′-8′′-biflavone family from *G. biloba* as potent TFEB activators. The successful application of this AI-driven drug discovery platform offers a promising strategy for developing effective drugs to treat neurodegenerative diseases.

Combining samples from patients with ALS and ALS iPSCs, we found lysosomal dysfunction in ALS motor neurons. This finding helps explain why multiple autophagy–lysosome pathway modulators, such as Trehalose [47] and Pridopidine [48] exhibited beneficial effects in ALS models. We confirmed that ISO promoted the nuclear translocation of TFEB by directly inhibiting GSK-3β and Lys85 within GSK-3β was a key site for ISO to competitively bind. This finding aligned with previously reported molecular dynamic simulation results showing that Lys85 was localized at the entrance of the activation pocket and played a key role in the insertion of ATP or substrate molecules [49], [50]. Lys85, as a key site for ATP-binding, was closely associated with GSK-3β activity [36], [37], [38]. Our results further demonstrated that Lys85 functioned as a "switch" for GSK-3β activity. The unique specific binding property of ISO, as a lead compound, offers a novel reference structure for developing next-generation GSK-3β inhibitors. Previous studies have extensively reported the protective effects of GSK-3β inhibitors, such as JGK-263 [51] and GSK-3β inhibitor VIII [52], in ALS animal models. However, the exact signaling pathway involved in this protective effect remains controversial [53]. Our study provides a novel insight into the mechanism underlying the beneficial effects of GSK-3β inhibition in ALS. Further validation of this mechanism in animal models of ALS is warranted.

While our study pioneers the combination of AI and wet-lab to identify novel TFEB agonists to treat ALS, we here acknowledge some limitations. First, the ligand-based model relied on existing known TFEB agonists, restricting the chemical diversity of hits and biasing predictions toward known structural scaffolds. Moreover, the integration with structure-based docking helped mitigate this limitation [54], but the approach still depended on the accuracy of predicted protein structures and docking algorithms [55]. Future refinements should involve incorporating generative AI models for *de novo* compound design, improving target conformational sampling, and integrating dynamic molecular simulations to enhance predictive reliability [56]. Second, although our data demonstrated lysosomal dysfunction in ALS motor neurons, the underlying molecular mechanisms remain unexplored. Third, whether ISO has additional biological targets and whether other novel mechanisms contribute to its neuroprotective effects in ALS motor neurons require further investigation in future studies. Finally, an iterative lead optimization process, including modification of its active moieties and structural optimization, is needed to enhance the therapeutic potential of ISO as a natural compound. Given the pleiotropic functions of GSK‑3β, chronic inhibition using pharmacological inhibitors like ISO may carry potential oncogenic risks. GSK‑3β negatively regulates multiple oncogenic proteins such as β‑catenin and c‑Myc [57]. Prolonged inhibition could therefore promote abnormal cell proliferation and increase the likelihood of tumorigenesis. For future clinical translation, the long‑term safety and tissue‑specific effects of ISO and its derivatives should be rigorously evaluated in appropriate preclinical models.

The ALS therapeutics approved by the U.S. FDA include Riluzole which counteracts excitotoxicity[58], and Edaravone, which is a small-molecule antioxidant [59]. However, their clinical benefits remain suboptimal, which is possibly due to the high heterogeneity of ALS and rapid disease progression [60]. Qalsody (tofersen) has also been approved for treating patients with ALS harboring *SOD1* gene mutations, yet it is limited by its genetic specificity [61]. TFEB agonists represent a bold, yet viable, approach to treating ALS. The use of TFEB agonists may serve as a pharmacological strategy for improving lysosomal function, which has been extensively validated across a range of diseases [62], [63]. Meanwhile, our ISO-derived data enable us to propose combining antioxidants and/or anti-excitotoxic agents with strategies enhancing lysosomal function specifically to clear damaged organelles and/or toxic aggregated proteins, yielding additive or even synergistic, therapeutic benefits in patients with ALS. Considering that lysosomal dysfunction represents a shared pathological hallmark across numerous neurodegenerative diseases and lysosomal storage disorders, ISO has substantial potential for clinical translation in treating these diseases in the future.

## 4 Materials and methods

### 4.1 Reagents and antibodies

The reagents used in this study were as follows: Isoginkgetin (HY-N2117; MCE; Dissolved in DMSO), Torin 1 (HY-13003; MCE), BI-D1870 (HY-10510; MCE), XAV-939 (HY-15147; MCE), LY294002 (HY-10108; MCE), Go6976 (HY-10183; MCE), ATP (HY-B2176; MCE), N2 (17502–048; Gibco), B27 (17504–044; Gibco), L-ascorbic acid (4055–50–81–7; Tocris Bioscience), CHIR-99021 (252917–06–9; Tocris Bioscience), DMH-1 (4126; Tocris Bioscience), SB431542 (1614; Tocris Bioscience), retinoic acid (04–0021; Stemgent), Purmorphamine (04–0009; Stemgent), valproic acid (VPA)(04–0007; Stemgent), compound E (6476; Tocris Bioscience), insulin-like growth factor (IGF) (HY-P7018; MCE), brain-derived neurotrophic factor (BDNF) (HY-P7116A; MCE), ciliary neurotrophic factor (CNTF) (HY-P7146; MCE), Y-27632 (S1049; SELLECKCHEM), and MG-132 (HY-13259; MCE).

Primary antibodies used in this study were as follows: anti-TFEB (37785, 1:1000; 91767, 1:100; Cell Signaling Technology), anti-LAMP2 (34141, 1:1000; Cell Signaling Technology), anti-cathepsin B (31718, 1:1000; Cell Signaling Technology), anti-cathepsin D (88239. 1:1000; Cell Signaling Technology), anti-GAPDH (AF0006, 1: 5000; Beyotime), anti-β-actin (AF0003, 1: 5000; Beyotime), anti-α-tubulin (2144, 1:2000; Cell Signaling Technology), anti-GFP (50430–2-AP, 1:1000; Proteintech), anti-rabbit IgG (30000–0-AP, 1:200; Proteintech), anti-GSK-3β (22104–1-AP, 1:1000; Proteintech), anti-mCherry (26765–1-AP, 1:2000; Proteintech), anti-histone H3 (AF0009, 1: 5000; Beyotime), anti-phospho-ULK1 (Ser757) (14202, 1:1000; Cell Signaling Technology), anti-phospho-p70 S6 Kinase (Thr389) (9205, 1:1000; Cell Signaling Technology), anti-phospho-4E-BP1 (Thr37/46) (2855, 1:1000; Cell Signaling Technology), anti-phospho-(Ser/Thr) Phe (9631, 1:1000; Cell Signaling Technology), anti-β-catenin (ab32572, 1:1000; Abcam), anti-phospho-β-catenin (Ser33/37/Thr41) (9561, 1:1000; Cell Signaling Technology), anti-c-Myc (18583, 1:1000; Cell Signaling Technology), anti-phospho-c-Myc (Thr58) (46650, 1:1000; Cell Signaling Technology), anti-HB9/HLXB9/MNX1 (ab92606, 1:500; Abcam), anti-MAP2 (ab318993, 1:5000; Abcam), and anti-PSA-NCAM-APC (130–120–437, 1:50; Miltenyi Biotec).

Secondary antibodies used in this study were as follows: anti-rabbit IgG, HRP-linked antibody (7074, 1:5000; Cell Signaling Technology), anti-mouse IgG, HRP-linked antibody (7076, 1:5000; Cell Signaling Technology), Alexa Fluor 488 goat anti-mouse IgG (A11029, 1:500; Invitrogen), Alexa Fluor 568 goat anti-rabbit IgG (A11011, 1:500; Invitrogen), Alexa Fluor 647 goat anti-rabbit IgG (A21235, 1:500; Invitrogen), and goat anti-chicken IgY H&L (Alexa Fluor 488) (ab150173, 1;5000; Abcam).

### 4.2 Postmortem samples from patients with ALS

Postmortem brain samples of patients with ALS and sex-matched healthy controls were obtained from the Netherlands Brain Bank (https://www.e-nbb.org). Sample details are available in Table S6. The related experiments were performed at Akershus University Hospital (Norway) and approved by the Regional Committee for Medicine and Health Research Ethics (REK #412997).

### 4.3 Cell lines, cell culture, and transfection

TFEB-EGFP HeLa cells, HeLa cells, and SH-SY5Y cells were cultured in DMEM high-glucose medium (11965092; Gibco) containing 10% fetal bovine serum (FBS, 10099–141; Gibco) and grown at 37℃ in the presence of 5% CO_2_. The point mutation was constructed using mutation-bearing primers via polymerase chain reaction (PCR) by Guangzhou IGE Biotechnology Co., Ltd. The cells were transfected with wild type TFEB-EGFP, TFEB S122D/S211D-EGFP (serine mutated to aspartic acid), TFEB S134D/S138D-EGFP (serine mutated to aspartic acid), Ub-R-GFP, Ub-G76V-GFP (glycine mutated to valine), GSK-3β-mCherry and its truncated expression plasmids (amino acids 1–55 corresponding to the GSK-3β’s N-terminal region, amino acids 55–340 forming its kinase domain, and amino acids 340–420 constituting its C-terminal region [64]), GSK-3β S9A (serine mutated to alanine), GSK-3β S9D (serine mutated to aspartic acid), GSK-3β K85A (lysine mutated to alanine), GSK-3β K85D (lysine mutated to aspartic acid), GSK-3β K85P (lysine mutated to proline), GSK-3β K85A-mCherry, GSK-3β K85D-mCherry, and GSK-3β K85P-mCherry mutant plasmids using Lipofectamine 3000 reagent (L3000001; Thermo Fisher Scientific).

Regarding the healthy control iPSC lines, UC12-iPSC was maintained in our laboratory, GZF2-iPSC was a gift from Dr. Xingguo Liu [42], UC01-iPSC was a gift from Dr. Kai Lei [43], and 2–8–8–2-iPSC was a gift from Dr. Dongwei Li [44]. Two iPSC lines from patients with ALS, which were purchased from WiCell Research Institute Inc., harbored the following mutations: TDP-43 (PFIZi013-A, *TARDBP A382T*) and SOD-1 (WC034i, *SOD1 D90A*). We generated isogenic controls. We introduced the *SOD1 G94A* mutation into the healthy UC12-iPSC line and introduced the *TARDBP A315T* mutation into the healthy GZF2-iPSC line. All the iPSC cells were cultured in Matrigel (354277; Corning)-precoated cell culture plastic plates or confocal dishes in mTeSR Plus basal medium (100–0276; STEMCELL Technologies) in an incubator at 37°C in the presence of 5% CO_2_. The culture medium was replaced daily.

The A315T point mutation (GCT to ACG) in *TARDBP* was introduced using the Alt-R CRISPR–Cas9 system [Integrated DNA Technologies (IDT)]. A guide RNA targeting exon 6 near codon 315 was designed using IDT’s CRISPR design tool. Synthetic crRNA (5’-ATGGGAGGAGGCATGAATTT-3’) and tracrRNA were annealed and complexed with Alt-R S.p. Cas9 Nuclease V3 to form ribonucleoprotein (RNP) complexes. A single-stranded oligodeoxynucleotide (ssODN) donor template (88-nt in length, 5’-CTGGGAAACAATCAAGGTAGTAATATGGGAGGAGGCATGAATTTTGGTACGTTCAGCATTAATCCAGCCATGATGGCTGCCGCCCAGGC-3’) harboring the A315T mutation and silent mutations disrupting unwanted substitutions was synthesized for homology-directed repair. Human healthy control GZF2-iPSCs were electroporated with RNP and ssODN using the Lonza 4D-Nucleofector system (program CA137). The cells were cultured in mTeSR medium with 10 µM Y-27632 for 24 h post-transfection. Genomic DNA was extracted 72 h later. The target region was then PCR-amplified (F: 5’-CGAACCTAAGCACAATAGCAATAGACAG-3’; R: 5’-TGCACCAGAATTAGAGCCACTATAAGAG-3’) and sequenced to confirm precise editing. Heterozygous clonal lines were generated by single-cell sorting and validated by Sanger sequencing.

The G94A point mutation (GGT to GCT) in *SOD1* was introduced via the Cas9-mediated knock-in method. Two guide RNAs targeting exon 4 (gRNA-A1: 5’-AATGTGACTGCTGACAAAGATGG-3’; gRNA-B1: 5’-GACTGCTGACAAAGATGGTGTGG-3’) were designed using the Zhang Lab’s CRISPR design tool at crispr.mit.edu and cloned into the PX459 vector containing Cas9. Human healthy control UC12-iPSCs were co-transfected with PX459 constructs and ssODNs encoding the G94A mutation using Lipofectamine Stem Transfection Reagent. The cells were selected with 1 µM puromycin after 72 h. Genomic DNA was extracted. The target region was PCR-amplified (F: 5’-GCCTAGCTACTTGTTTGCAAATTTG-3’; R: 5’-CAAGTGAGAAACCCAATCCTGGC-3’) and sequenced to confirm precise editing. Heterozygous clonal lines were generated by single-cell sorting and validated by Sanger sequencing.

### 4.4 Ligand-based virtual screening

LBVS was initially performed using the DDD platform (https://deepdrugdiscovery.mindrank.ai/). A total of 15 known mTOR-independent TFEB nuclear translocation agonists were used as query ligands (Table S1). The DDD system integrated 1D, 2D, and 3D molecular features through advanced attention-based molecular representations, thereby enabling rapid similarity-based selection from large libraries [15]. Candidate compounds were ranked according to DDD similarity scores. Those with similarity values greater than 0.65 were retained for further analysis, following the established selection criteria described in the DDD platform. Based on these parameters, 1745 candidate compounds with similar molecular features from a large compound library, gathered and curated from commercial sources (totaling 1155,606 molecular compounds; Topscience and MCE), were prioritized and subsequently advanced for subsequent structure-based virtual screening and docking validation.

### 4.5 Receptor preparation

Protein structures related to the autophagy–lysosome pathway were collected from GeneCards (https://www.genecards.org/) for reverse virtual screening. All relevant UniProt identifiers were obtained from the UniProt Knowledgebase (https://www.uniprot.org/). The corresponding structural models were retrieved from the AlphaFold Protein Structure Database (AlphaFold2, https://alphafold.com/). A total of 1028 protein structures were collected. Among these, 22 entries lacked structural models due to the presence of noncanonical residues or incompatible sequence features. All analyzable protein structures (*n* = 1006) were processed using PDBFixer (v1.11) to repair missing atoms, add hydrogen atoms, and assign protonation states at a physiological pH of 7.4.

### 4.6 Structure-based screening

Binding pockets were predicted using P2Rank (v2.3), which is a tool that ranks potential sites based on surface geometry and physicochemical properties [19]. Proteins without predicted binding pockets or those directly linked to the mTOR signaling pathway were excluded (*n* = 98). For the remaining 908 proteins, the top-ranked pocket per protein was selected for docking analysis. Docking simulations were conducted using Uni-Dock (v1.1.1), a GPU-accelerated docking engine employing the Vina scoring function and a Monte Carlo–based global search algorithm [20]. Each docking grid was centered on the predicted pocket and defined as a 25 × 25 × 25 Å³ cubic box to ensure complete coverage of the binding pocket.

### 4.7 Post-docking analysis

The workflow generated a 1745 × 908 cross-docking score matrix. For each candidate compound, the mean of its top 20 docking scores across all proteins was calculated and defined as the final binding score. Compounds with mTOR protein-binding affinity scores less than 8.5 were excluded to identify candidates not interfering with the mTOR signaling pathway. Based on this ranking by this final binding score, the top 20 compounds were selected as final candidate compounds for subsequent biological experimental validation.

### 4.8 Code, hardware, and runtime

Code and processed data to reproduce all analyses are publicly available at: https://github.com/XiangLuXiao/mTORIndependentCompoundScreen

The repository provides the complete computational workflow and scripts used in this study, together with the corresponding processed datasets (including docking score matrices and final prioritized compounds). Access is fully open and does not require any login or personal information.

All virtual screening computations were performed on a high-performance server equipped with eight Intel Xeon Platinum 8358 @ CPUs (2.60 GHz) and one NVIDIA L40S GPU (48 GB). Protein structure download and preparation took 1 min 27 s and 7 min 21 s, respectively. Pocket prediction was conducted in approximately 6 min, and structure-based molecular docking required ∼18 h in total.

### 4.9 Molecular docking experiments

The SwissTargetPrediction online tool (http://www.swisstargetprediction.ch/) was employed to identify the possible molecular targets of ISO. The SMILES of isoginkgetin was obtained from the PubChem (https://pubchem.ncbi.nlm.nih.gov/). The CB-Dock2 online tool was used (https://cadd.labshare.cn/cb-dock2/php/index.php) with auto blind docking to perform a molecular docking simulation study of ISO with GSK-3β protein. The crystal structure of GSK-3β in complex with its ligand (PDB ID: 4J1R) was obtained from the Protein Data Bank (http://www.rcsb.org). The 3D structure of ISO was constructed using ChemBioDraw software (Oxford, UK). Solvent molecules and ligands were removed using PyMoL software to prepare the docking components for later molecular docking. The 3D structures with the docked configurations of the lowest binding energy (Δ*G*) were visualized using PyMoL software.

### 4.10 Immunoblotting

After the designed treatments, the harvested cells were lysed with Laemmli SDS buffer (62.5 mM Tris-HCl, pH 6.8, 25% glycerol, 2% SDS, phosphatase inhibitor, and proteinase inhibitor cocktail; 78446, Thermo Fisher Scientific). The nuclear–cytoplasmic fractionation experiment was performed using the cell nucleus protein and cytoplasmic protein extraction kit (P0027; Beyotime). An equal amount of protein was resolved using SDS-PAGE and transferred to a PVDF membrane (1620177; Bio-Rad Laboratories). The membrane was blocked with 5% skimmed milk (1706404; Bio-Rad Laboratories) for 1 h, probed with primary and secondary antibodies, developed with the enhanced chemiluminescence kit (WBKLS0500; Millipore), and visualized with the Bio-Rad ChemiDoc Imaging System.

### 4.11 Immunoprecipitation

The total proteins were extracted from TFEB-EGFP HeLa cells using cell lysis buffer for Western blotting and IP (Beyotime, P0013) with protease and phosphatase inhibitors. Protein A/G magnetic beads (HY-K0202; MCE) were incubated with GFP antibody [dilution 1:50 (*v*/*v*), 50430–2-AP, Proteintech] or rabbit IgG antibody (30000–0-AP; Proteintech) at room temperature for 1 h. Then, protein lysate was added and incubated overnight at 4°C. The immunoprecipitation complex was collected using both a magnetic rack and centrifugation and washed with cell lysis buffer with protease and phosphatase inhibitors. The mixture was analyzed by Western blotting.

### 4.12 Thermal proteome profiling

The TPP assay was conducted as previously reported [65]. Briefly, the protein samples extracted from SH-SY5Y cells were divided into two parts and treated with DMSO (as a control) and ISO, respectively. Then, each sample was divided into 10 aliquots, which were subjected to heating at different temperatures (from 37 °C to 67 °C). After digestion with trypsin, conventional data-dependent acquisition mass spectrometry was used to establish and analyze a spectrum of proteins obtained from samples treated at 37 °C. Subsequently, we used the data-independent acquisition method to collect mass spectrometry data for each sample. Based on protein quantification data, the melt curves of each protein were fitted using the Bioconductor TPP package and the melting point was calculated. Then, the differences in melting point between the DMSO and the ISO-treated groups were compared.

### 4.13 Cellular thermal shift assay

HeLa and SH-SY5Y cells were treated with ISO (50 μM) or DMSO for 8 h. After treatment, the cells were washed with phosphate-buffered saline (PBS) containing a protease inhibitor (1 mmol/L PMSF), harvested, and evenly aliquoted into seven PCR tubes. The cells in each tube were incubated at seven different temperatures (37 °C, 41 °C, 45 °C, 49 °C, 53 °C, 57 °C, and 61 °C) for 3 min in a thermal cycler (T100; Bio-Rad). Subsequently, the cells were quickly frozen in liquid nitrogen and lysed on ice using NP40 buffer to extract total proteins. The samples were then centrifuged at 20,000 *g* and 4 ℃ for 10 min. The supernatant was harvested and mixed with loading buffer for subsequent Western blotting.

### 4.14 Protein truncation test

For mammalian expression, the full-length or truncated *GSK-3β* gene from *Homo sapiens* was inserted into the *Bam*HI/*Age*I restriction site of pFUGW-mCherry vector (Plasmid #131505; Addgene). The ligation products were transfected into DH5α competent cells and selected on ampicillin plates. Positive clones were validated by colony PCR and Sanger sequencing. Endotoxin-free plasmid DNA was prepared using Qiagen Plasmid Kits. Different constructs were transfected into TFEB-EGFP HeLa cells using Lipofectamine 3000 transfection reagent (L3000001; Thermo Fisher Scientific). Then, ISO was added to the culture medium to investigate the changes in the ability of ISO to induce TFEB nuclear translocation. The expression of full-length or truncated GSK-3β-mCherry fusion proteins was confirmed via Western blotting with anti-GSK-3β (22104–1-AP; Proteintech) and anti-mCherry (26765–1-AP; Proteintech) antibodies.

### 4.15 Protein expression and purification

A gene of wild-type *GSK-3β* (Gene ID: 2932; NCBI) from *H. sapiens* was codon-optimized and synthesized. First, the gene was inserted into the *Eco*RI/*Xho*I restriction site of the pET-28a vector to express the protein fused with 6x His tag at the C-terminus. Using the same method, plasmids encoding three GSK-3β mutant forms (K85P, Y134P, and D200P; each amino acid mutated to proline) were constructed. *Escherichia coli* BL21(DE3) cells harboring the GSK-3β expression plasmid were grown at 37°C in 4 L of Luria–Bertani medium containing 100 μg/mL kanamycin. When the culture reached OD600 of 0.6–0.8, 0.2 mM IPTG was added to the medium and incubated further at 20°C for 12 h. The cells were harvested by centrifugation at 5000 *g* for 15 min and frozen.

His tag protein purification kit (IDA-Ni agarose magnetic beads, P2245; Beyotime) was used for protein purification. The frozen cells were resuspended in lysis buffer and then disrupted by sonication, following the manufacturer’s protocols. The insoluble materials were removed by centrifugation at 20,000 *g* for 10 min. The supernatant was mixed with IDA-Ni agarose magnetic beads and washed five times with the wash buffer provided in the kit. Finally, the elution was performed using the magnetic rack to obtain the target protein. For each operation, 10 μL of the sample was retained and added to the loading buffer for denaturation, which was then used for Western blotting detection. The remaining proteins were subjected to protein dialysis using regenerated cellulose dialysis membranes (25 mm, 14 kDa, FDM214; Beyotime). The proteins were dissolved in the ITC buffer (25 mM HEPES, 75 mM NaCl, and 0.5 mM EDTA, pH 7.5) for subsequent experiments.

### 4.16 Isothermal titration calorimetry

ITC measurements were performed on a MicroCal-ITC microcalorimeter (Malvern Panalytical, Malvern, UK). Isothermal calorimetric titration experiments were performed at 25 ℃ with purified protein of GSK-3β and its mutants and ISO dissolved in ITC buffer (25 mM HEPES, 75 mM NaCl, and 0.5 mM EDTA, pH 7.5, and 2% DMSO). For ligand–protein binding, 40 μL of ISO (300 μM, 2% DMSO) was injected 19 times from a stirring syringe into a cuvette containing about 300 μL of the protein (15 μM, 2% DMSO). The first injection volume was 0.2 μL, followed by 19 injections of 2 μL, with a duration of 40 s; the time interval between injections was 150 s. ITC buffer (2% DMSO) was used to titrate the GSK-3β protein (15 μM, 2% DMSO) as a control under the same experimental conditions. All the analyses were conducted using the MicroCal PEAQ-ITC Analysis software.

### 4.17 Kinetic analysis on GSK-3β

The GSK-3β kinase activity assay was performed using a commercial GSK-3β kinase enzyme system (V1992; Promega). First, the enzyme, substrate, ATP, and inhibitor (ISO, 10 μM) were diluted in the kinase buffer solution following the manufacturer’s protocols, using CHIR-99021 (1 μM, HY-10182; MCE) as the positive control. The ADP-Glo Kinase Assay system was used after incubating the mixture at room temperature for 60 min. The kinase activity of GSK-3β was determined by measuring the luminescence value with a fluorescence microplate reader.

The IC_50_ (concentration at which 50% of enzyme inhibition occurs) value was determined using the same method. In the first step, different concentration gradients of the inhibitor ISO were added into the kinase buffer solution (0, 0.1, 1, 5, 10, 15, 25, 30, 100, 1000 μM). The kinase activity of GSK-3β was detected using the ADP-Glo Kinase Assay system. The IC_50_ value of ISO was calculated using GraphPad Prism 8.0.

The protocol of the kinetic experiments was similar to that of the GSK-3β inhibition tests. The kinetic activity of GSK-3β (1.56 ng) was measured at 10 and 15 μM ISO separately. The relationship between the ISO and ATP was tested by keeping the concentration of substrate glycogen synthase 1 (GS-1, amino acid 636–661) unchanged at 0.02 μg/well, while setting the concentration of ATP at 25, 50, and 100 μM. A Lineweaver–Burk plot using GraphPad Prism 8.0 was developed by plotting 1/*v* (the inverse of *v*, where *v* was obtained from the kinase activity test) versus 1/[ATP] (the inverse of the ATP concentration) to determine whether it was the ATP-competitive binding and to calculate the kinetic parameters [66].

### 4.18 Immunofluorescence and DNA staining

iPSC-derived motor neurons were grown in 35-mm confocal glass-bottom dishes (NEST Biotechnology), fixed with 4% paraformaldehyde (PFA) at room temperature for 15 min, and then incubated in blocking buffer [1% bovine serum albumin (BSA), 0.1% Triton X-100 in PBS] for 1 h. After blocking, the cells were incubated with the primary antibody overnight at 4°C and washed three times with PBS at room temperature for 10 min. The cells were further incubated with the fluorochrome-conjugated secondary antibody at room temperature for 60 min and then washed three times with PBS at room temperature for 10 min. The cell nuclei were stained with 4’,6-diamidino-2-phenylindole (DAPI; 1:2000) and visualized using a Zeiss LSM800 confocal laser scanning microscope.

### 4.19 iPSC-derived motor neurons

Motor neurons were generated from iPSCs as described previously [67]. iPSCs were dissociated with Accutase (07922; Stemcell) and split 1:6 on Matrigel-coated plates with mTeSR (containing 10 μM Y-27632). On the following day, the medium was replaced with a chemically defined neural medium containing DMEM/F12 (C11330500BT; Gibco), neurobasal medium (21102–049; Gibco) in a ratio of 1:1, 0.5 × N2 ( 17502–048; Gibco), 0.5 × B27 (17504–044; Gibco), 0.1 mM L-ascorbic acid (4055–50–81–7; Tocris Bioscience), and 1 × GlutaMAX (35050061; Gibco). 3 μM CHIR-99021 (252917–06–9; Tocris Bioscience), 2 μM DMH-1 (4126; Tocris Bioscience), and 2 μM SB431542 (1614; Tocris Bioscience) were added to the medium. The culture medium was changed every day. Human iPSCs were maintained under these conditions for 6 days and induced into neuroepithelial (NEP) cells. The NEP cells were then dissociated with Accutase and split in a ratio of 1:6 with the same medium as described earlier. Then, 0.1 μM retinoic acid (RA) (04–0021; Stemgent), 0.5 μM purmorphamine (04–0009; Stemgent), 1 μM CHIR-99021, 2 μM DMH-1, and 2 μM SB431542 were added. The culture medium was changed on a daily basis. The NEP cells were maintained under these conditions for 6 days and differentiated into OLIG2^+^ motor neuron precursors (MNPs). The OLIG2^+^ MNPs were expanded in the same medium containing 3 μM CHIR-99021, 2 μM DMH-1, 2 μM SB431542, 0.1 μM RA, 0.5 μM purmorphamine, and 0.5 mM VPA (04–0007; Stemgent) and split in a ratio of 1:6 once weekly with Accutase. OLIG2^+^ MNPs were frozen in 70% DMEM/F12, 20% FBS, and 10% DMSO in liquid nitrogen until use. After thawing, the cells grew in expansion medium.

OLIG2^+^ MNPs were dissociated with Accutase and cultured in suspension in neural medium containing 0.5 μM RA and 0.1 μM purmorphamine to induce motor neuron differentiation. The culture medium was changed daily. OLIG2^+^ MNPs were differentiated into motor neurons after 6 days. Finally, HB9^+^/MAP2^+^ motor neurons were cultured with 0.5 μM RA, 0.1 μM purmorphamine, 0.1 μM compound E (6476; Tocris Bioscience), and three neurotrophic factors including IGF (10 ng/mL, HY-P7018; MCE), BDNF (10 ng/mL, HY-P7116A; MCE), and CNTF (10 ng/mL, HY-P7146; MCE) for 10 days. During differentiation, Y-27632 (S1049; SELLECKCHEM) was added for 12 h during each passage. Neurotrophic factors were removed from the culture medium containing mature HB9^+^/MAP2^+^ motor neurons on day 21 to promote the appearance of ALS-related phenotypes [22].

### 4.20 Magnetic microbead sorting of neurons

Consistent with the previously reported method [45], OLIG2^+^ MNPs were dissociated with Accutase and blocked with a solution containing PBS, 0.5% BSA, and 2 mM EDTA. The cells were then incubated with PSA-NCAM-APC antibody (130–120–437; Miltenyi Biotec) for 10 min at 4 °C. They were washed and then incubated with anti-APC microbeads for 15 min at 4 °C. Subsequently, they were washed twice and filtered prior to loading into the separation column (LS column) attached to a magnetic stand (all from Miltenyi Biotec). After three rounds of washing, the column was removed from the magnetic stand, and the labeled cells were eluted in culture media for the subsequent differentiation of motor neurons.

### 4.21 Labeling of motor neurons and detection of lysosomal function

The pAAV-hSyn-EGFP vector (50465; Addgene) and pFUGW-mCherry lentivirus vector (131505; Addgene) were purchased from Addgene. The packaging and concentration of adeno-associated viruses and lentiviruses were performed by Guangzhou IGE Biotechnology Co., Ltd. For the pAAV-hSyn-EGFP AAV transfection experiment, 100,000 MNPs were seeded per confocal dish and continuously differentiated to obtain mature motor neurons. The pAAV-hSyn-EGFP AAV was transfected into motor neurons at an MOI of 50 per dish overnight. The cells were washed twice with culture medium, incubated for 72 h, and then allowed to differentiate into mature *Syn*::EGFP^+^/HB9^+^/MAP2^+^ motor neurons. On day 28, the lysosomal function in the labeled motor neurons was detected using DQ-BSA-Red (HY-D2449; MCE) staining, whereas the lysosome number in the labeled motor neurons was detected using LysoTracker Red (C1046; Beyotime) staining through live-cell imaging. The average fluorescence intensity of DQ-BSA-Red staining was analyzed using ImageJ software to indicate lysosomal function. The fluorescent area of LysoTracker Red staining was analyzed using ImageJ software to reflect the lysosome number. Consistently, after ISO treatment, the alterations in lysosomal function and lysosome number were detected using the same methods.

For the pFUGW-GSK-3β-mCherry lentiviral transfection experiment, 1,000,000 MNPs sorted using magnetic beads were inoculated into each well of the six-well plate and continuously differentiated to obtain mature motor neurons. The pFUGW-GSK-3β-mCherry lentivirus was transfected into motor neurons at an MOI of 50 per well overnight in the presence of polybrene (10 μg/mL, HY-112735; MCE). Then, the neurons were washed twice with the culture medium and cultured for 72 h to obtain motor neurons with overexpressed GSK-3β proteins. The transfected motor neurons were treated with ISO. The changes in lysosomal proteins were detected by Western blotting.

### 4.22 Live-cell imaging

The transfected living cells or cells treated with commercial dyes (DQ-BSA-Red or LysoTracker Red), or *Syn*::EGFP reporter system–labeled motor neurons were cultured in 96-well plates or 35-mm confocal dishes (NEST Biotechnology) at 37 °C in the presence of 5% CO_2_. Imaging was performed using the Operetta CLS PerkinElmer high-content imaging system or Zeiss LSM800 confocal microscope with live-cell station. The images were processed and analyzed using the ImageJ software.

### 4.23 Lactate dehydrogenase cytotoxicity assay

A total of 10,000 MNPs sorted using magnetic beads were seeded per well in a 96-well plate and continuously differentiated to obtain mature motor neurons. The culture medium was replaced with 200 μL of fresh media on day 28. The culture medium was then collected on day 35 after 7 days of ISO treatment. The motor neurons with GSK-3β overexpression were also treated by the same method to evaluate the protective effect of ISO on ALS motor neurons. CyQUANT LDH Cytotoxicity Assay (C20301; Invitrogen) was used for quantifying LDH leakage. The LDH leakage was recorded, normalized, and quantified following the manufacturer’s protocols.

### 4.24 Graphics

Graphics including graphical abstract, Fig. 3A, Fig. 5C, and Figure S2A were created with BioRender.com.

### 4.25 Statistical analysis

Blinding was implemented during the experimental procedures and data analysis of efficacy assessments in ALS iPSC-derived motor neurons, including LDH leakage, neurite morphology, and motor neuron survival. Investigators and data analysts were blinded to group allocation throughout the study to improve the reliability of experimental data. Data were presented as mean ± standard deviation, unless otherwise specified. The unpaired *t* test was used for comparison between two independent samples. One-way analysis of variance (ANOVA) was used for comparison among multiple groups, followed by Dunnett's multiple comparison test. Group differences were analyzed with two-way ANOVA, followed by Tukey’s multiple comparison test. GraphPad Prism 9.5 was used for statistical analysis. The significance level was indicated in all statistical analyses. *P* values less than 0.05 indicated statistically significant differences.

## CRediT authorship contribution statement

**Shu-qin Cao:** Writing – original draft, Investigation. **Chunzuan Xu:** Validation, Methodology. **Jing Wen:** Methodology, Investigation. **Krinos Li:** Methodology, Investigation. **Guopan Liu:** Methodology, Investigation. **Shenglong Deng:** Investigation, Formal analysis. **Yixia Ling:** Methodology, Investigation. **Huanxing Su:** Writing – review & editing, Supervision, Funding acquisition, Data curation, Conceptualization. **Guang Yang:** Project administration, Methodology, Funding acquisition. **Guang Lu:** Writing – review & editing, Validation, Supervision, Formal analysis. **Xianglu Xiao:** Methodology, Investigation, Formal analysis. **Dajiang Qin:** Writing – review & editing, Visualization, Project administration, Funding acquisition. **Ang Li:** Writing – original draft, Methodology, Investigation, Data curation, Conceptualization. **Fang Evandro Fei:** Writing – review & editing, Validation, Supervision, Project administration, Funding acquisition.

## Declaration of Competing Interest

The authors declare the following financial interests/personal relationships which may be considered as potential competing interests. E.F.F. is co-owner of Fang-S Consultation AS (Organization number 931 410 717) and NO-Age AS (Organization number 933 219 127). E.F.F also has an MTA with LMITO Therapeutics Inc (South Korea), a CRADA with ChromaDex (USA), a commercialization agreement with Molecule AG/VITADAO, and is a consultant to MindRank AI (China), NYO3 (Norway), and AgeLab (Vitality Nordic AS, Norway). S.Q.C. has a commercialization agreement with Molecule AG/VITADAO. X.L.X. is a consultant for Mindrank AI. S.L.D. is a consultant for Mindrank AI. The other authors have no relevant financial or non-financial interests to disclose.

## Data Availability

Data will be made available on request. All raw data for the paper can be downloaded via the WeTransfer link.all the original data

## Footnotes

Appendix A Supplementary data associated with this article can be found in the online version at doi:10.1016/j.phrs.2026.108172.
