# Supplementary material for: Isoginkgetin protects against degeneration of ALS motor neurons via regulating the GSK-3β–TFEB signaling axis
Source: Pharmacol Res. 2026 May;227:108172. doi: 10.1016/j.phrs.2026.108172 (PMC13132972; doi:10.1016/j.phrs.2026.108172)
Supplement: Supplementary file 2 — Supplementary material [file mmc2.docx]

| **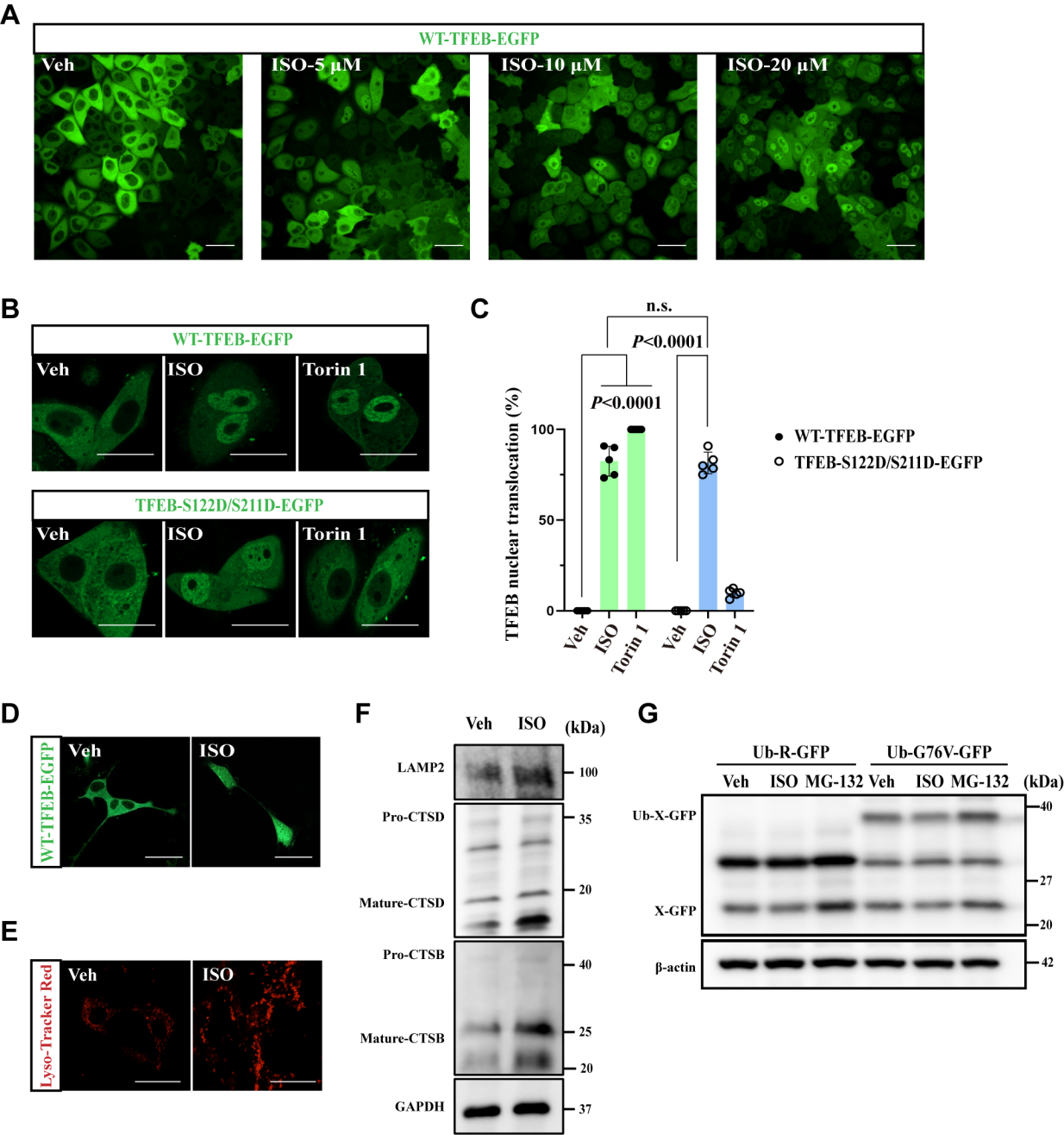** |
| --- |

**Figure S1. ISO promotes mTORC1-independent TFEB nuclear translocation and enhances lysosomal biogenesis and function.**

1. Live-cell imaging showed that ISO promoted the nuclear translocation of TFEB in a dose-response manner. Scale bars, 50 μm. (B-C) Following transfection of HeLa cells with plasmids encoding wild-type TFEB-EGFP and the TFEB S122D/S211D-EGFP mutant, treatment with ISO (10 μM, 12 h) promoted nuclear translocation of both TFEB variants. Scale bars, 50 μm. *n* = 5, five biological replicates. (D) After transfection of the wild type TFEB-EGFP plasmid into SH-SY5Y cells, treatment with ISO (10 μM, 12 h) promoted the nuclear translocation of TFEB. Scale bars, 50 μm. (E) ISO increased the number of functional lysosomes (indicated by LysoTracker Red staining) in SH-SY5Y cells. Scale bars, 25 μm. (F) Western blotting results showed that ISO increased the expression of lysosome-related proteins in SH-SY5Y cells. (G) After transfecting SH-SY5Y cells with Ub-R-GFP and Ub-G76V-GFP plasmids, Western blotting results showed no accumulation of these fusion proteins. This indicated that ISO did not inhibit UPS activity; MG-132 served (250 nM, 4 h) as a positive control. Two-way ANOVA was followed by Tukey’s multiple comparison test in (C).

| **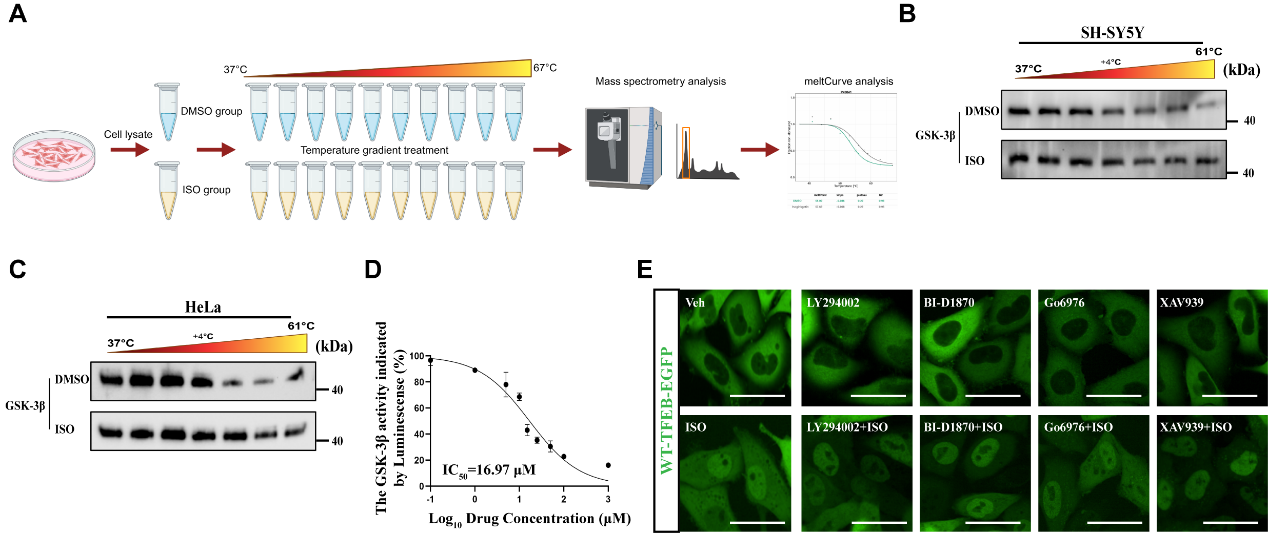** |
| --- |

**Figure S2. GSK-3β is identified as the molecular target of ISO.**

(A) Schematic diagram of TPP. (B) CESTA results showed that ISO (50 μM) increased the thermal stability of GSK-3β across a temperature gradient in SH-SY5Y cells. (C) CESTA results showed that ISO increased the thermal stability of GSK-3β across a temperature gradient in HeLa cells. (D) Detection using an *in vitro* kinase activity kit showed that the IC_50_ value of ISO for GSK-3β kinase activity was 16.97 μM; *n* = 5, five biological replicates. (E) TFEB-EGFP HeLa cells were co-treated with ISO (10 μM) and each of the following inhibitors for 12 h: PI3K/Akt inhibitor LY294002 (10 μM), MAPK/p90RSK inhibitor BI-D1870 (1 μM), pan-PKC inhibitor Go6976 (1 μM), and Wnt/β-catenin pathway inhibitor XAV939 (1 μM). The results demonstrated that ISO still promoted TFEB nuclear translocation, thereby ruling out the possibility that ISO influenced GSK-3β activity through other signaling pathways. Scale bars, 50 μm.

| 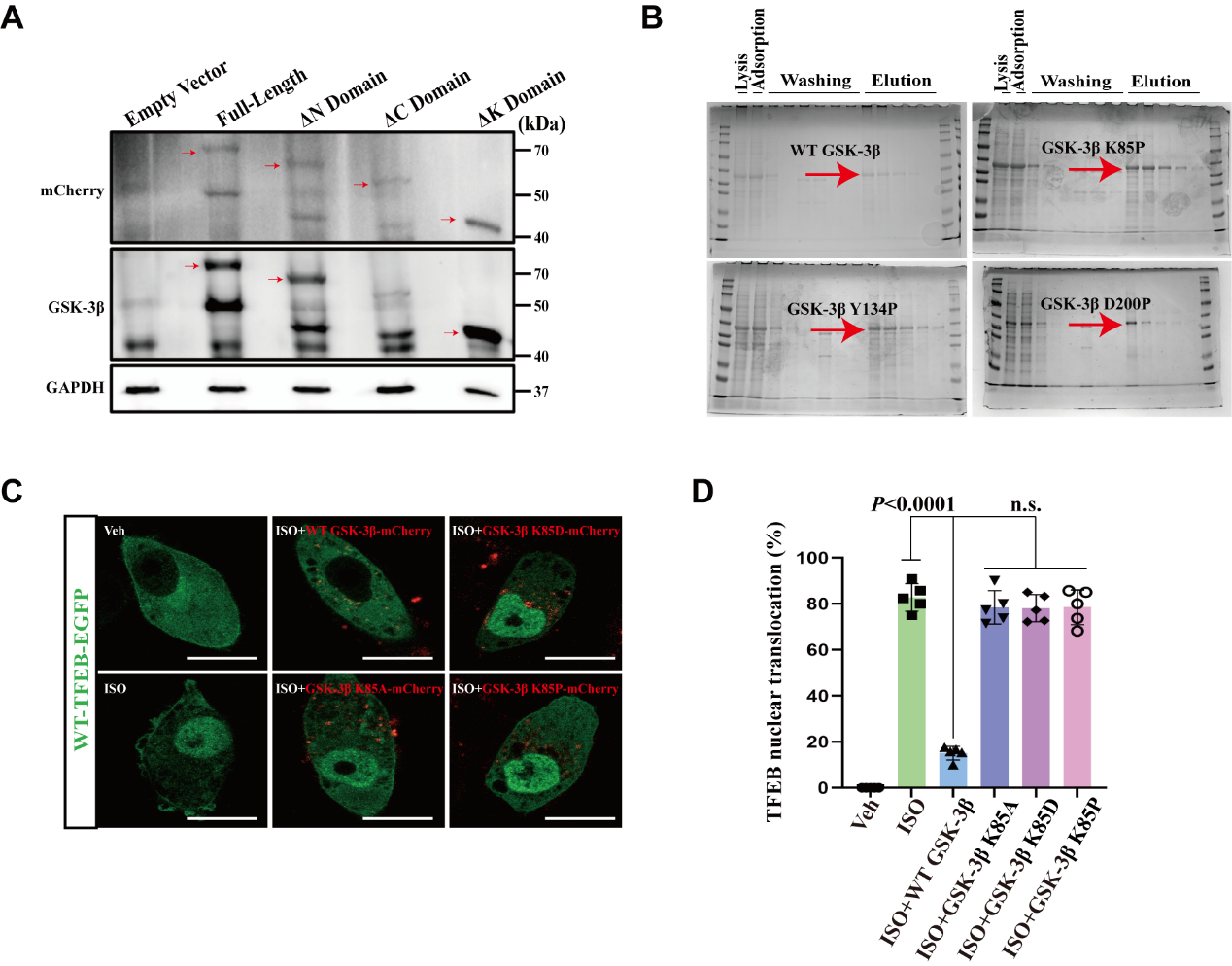 |
| --- |

**Figure S3. Lys85 residue of GSK-3β represents a crucial site for ISO action.**

(A) Western blotting results showed that the full-length GSK-3β-mCherry fusion protein and its truncated forms were expressed in TFEB-EGFP HeLa cells (red arrow). Since the GSK-3β antibody recognized a specific epitope in the C-terminal domain of the GSK-3β protein (amino acids 355–433; <https://www.ptgcn.com/products/GSK3B-Antibody-22104-1-AP.htm>), the C-terminally truncated forms of GSK-3β could not be detected. (B) Coomassie Brilliant Blue staining results showed that the wild-type GSK-3β and its three mutant proteins (GSK-3β K85P, GSK-3β Y134P, and GSK-3β D200P) were successfully purified (red arrow). (C and D) Live-cell imaging results showed that three Lys85 residue mutants of GSK-3β (GSK-3β K85A-mCherry, GSK-3β K85D-mCherry, and GSK-3β K85P-mCherry) failed to block ISO-induced TFEB nuclear translocation; *n* = 5, five biological replicates. Scale bars, 25 μm. Data were presented as the mean ± standard deviation. One-way ANOVA was followed by Dunnett's multiple comparison test.

| 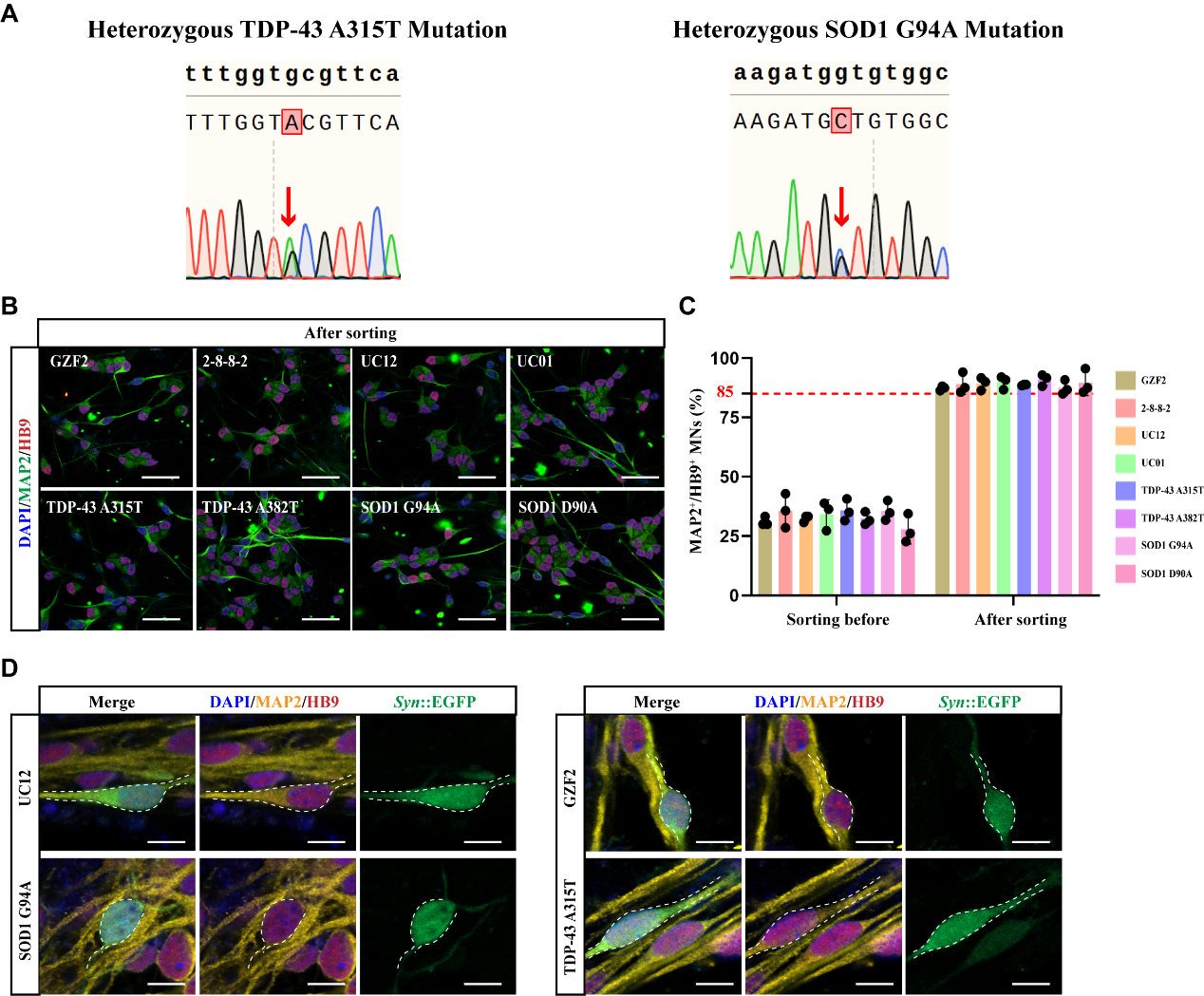 |
| --- |

**Figure S4. Purification and live-cell labeling of motor neurons.**

(A) Sanger sequencing confirmed heterozygous mutations: TDP-43 (p.A315T) in GZF2-iPSCs and SOD1 (p.G94A) in UC12-iPSCs. (B) Representative images of HB9⁺/MAP2⁺ motor neuron differentiation on day 10 following magnetic microbead sorting of neurons. Scale bars, 100 μm. (C) Quantitative analysis of the proportion of HB9⁺/MAP2⁺ motor neurons before and after magnetic microbead sorting revealed that sorting increased this proportion to more than 85% across motor neurons derived from eight iPSC lines; *n* = 3, three biological replicates. (D) Representative immunofluorescence staining images showed that the neuronal reporter system *Syn*::EGFP could label HB9⁺/MAP2⁺ motor neurons on day 21. Scale bars, 10 μm. Data were presented as the mean ± standard deviation.


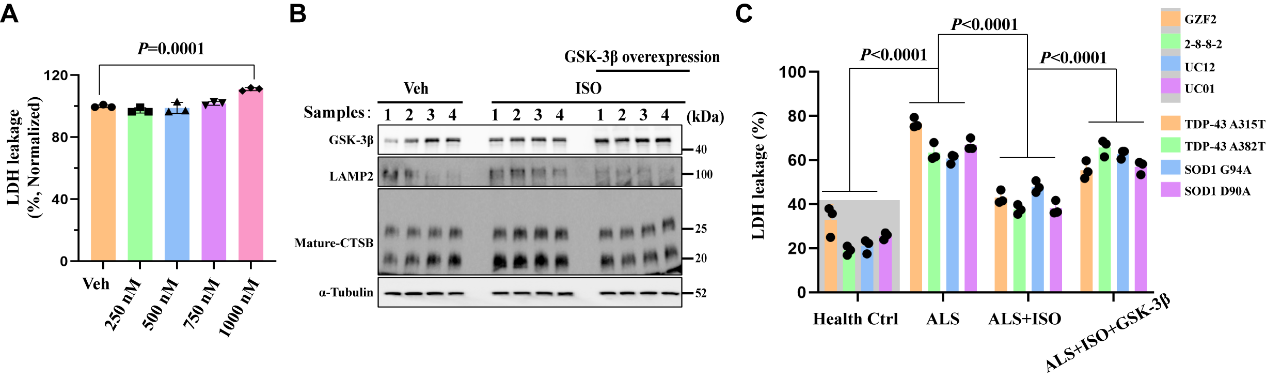


**Figure S5.** **Overexpression of GSK-3β in ALS motor neurons blocks the beneficial effects of ISO.**

(A) In the healthy control UC-12-iPSC-derived motor neurons, the LDH release assay showed that treatment with 750 nM ISO exhibited no significant neurotoxicity; *n* = 3, three biological replicates. (B) Western blotting results showed that GSK-3β overexpression blocked the ISO-induced upregulation of lysosome-related protein expression in four types of motor neurons. Sample 1: TDP-43 A315T; Sample 2: TDP-43 A382T; Sample 3: SOD1 G94A; and Sample 4: SOD1 D90A. (C) LDH leakage results showed that ISO reduced LDH leakage from four types of ALS motor neurons, whereas GSK-3β overexpression blocked this beneficial effect; *n* = 3, three biological replicates. Data were presented as the mean ± standard deviation. One-way ANOVA was followed by Dunnett's multiple comparison test in (A). Two-way ANOVA was followed by Tukey’s multiple comparison test in (C).
