# Supplementary material for: Isoginkgetin protects against degeneration of ALS motor neurons via regulating the GSK-3β–TFEB signaling axis
Source: Pharmacol Res. 2026 May;227:108172. doi: 10.1016/j.phrs.2026.108172 (PMC13132972; doi:10.1016/j.phrs.2026.108172)
Supplement: Supplementary file 3 — Supplementary material [file mmc3.docx]

| **Supplementary Table 1 mTOR-independent TFEB agonists** | | |
| --- | --- | --- |
| **Compound** | **Structure** | **Reference** |
| Sulforaphane | 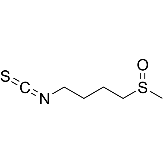 | [1] |
| Kaempferide | 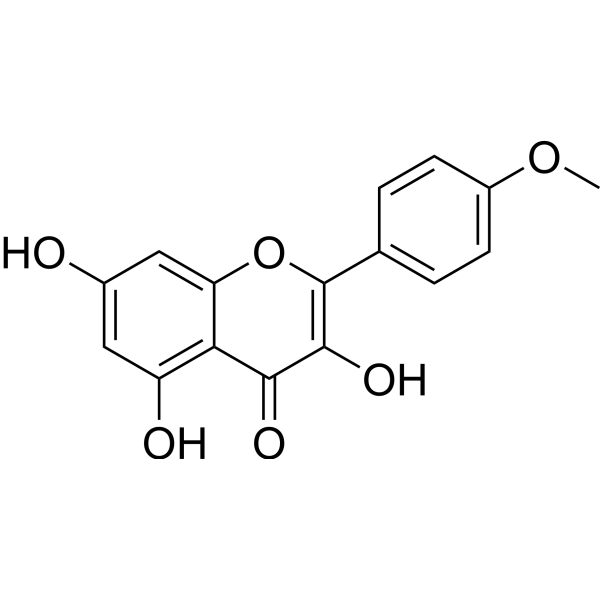 | [2] |
| SB202190 | 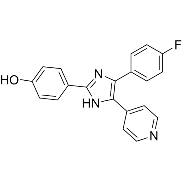 | [3] |
| Trigonochinene E | 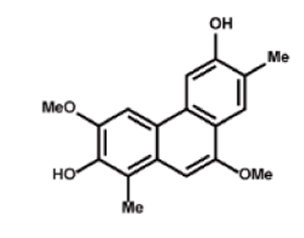 | [4] |
| Trehalose | 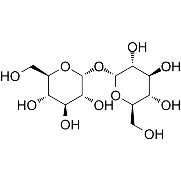 | [5] |
| Akebia saponin E | 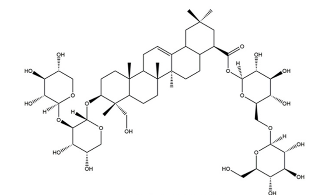 | [6] |
| Ezetimibe | 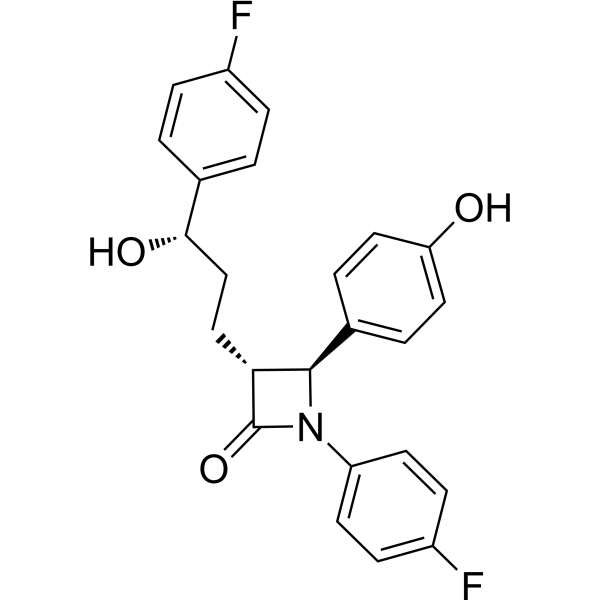 | [7] |
| Acacetin | 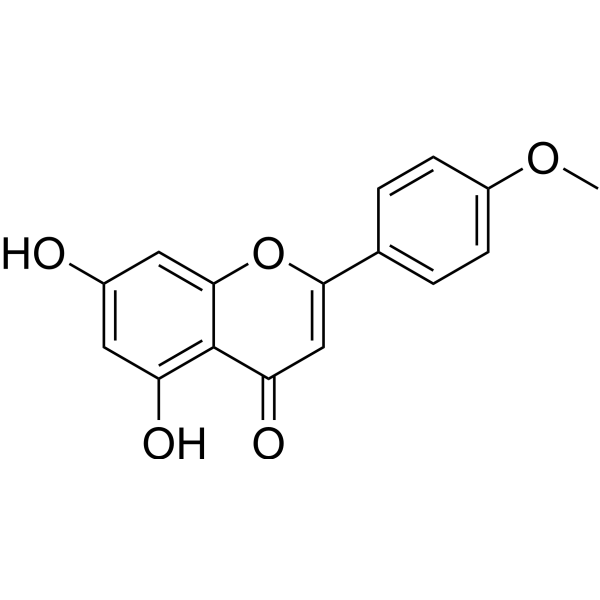 | [8] |
| HEP14 | 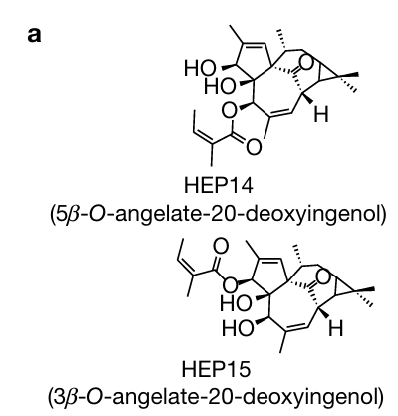 | [9] |
| HEP15 | 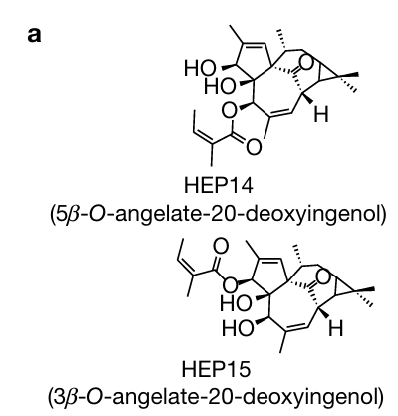 | [9] |
| Abemaciclib | 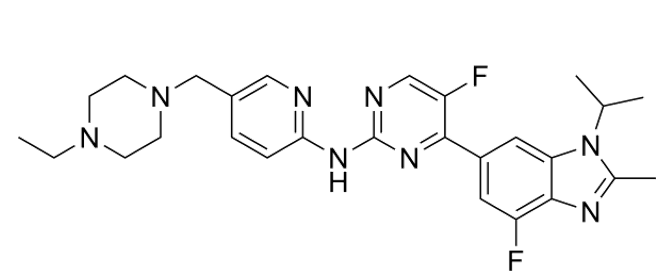 | [10] |
| Palbociclib | 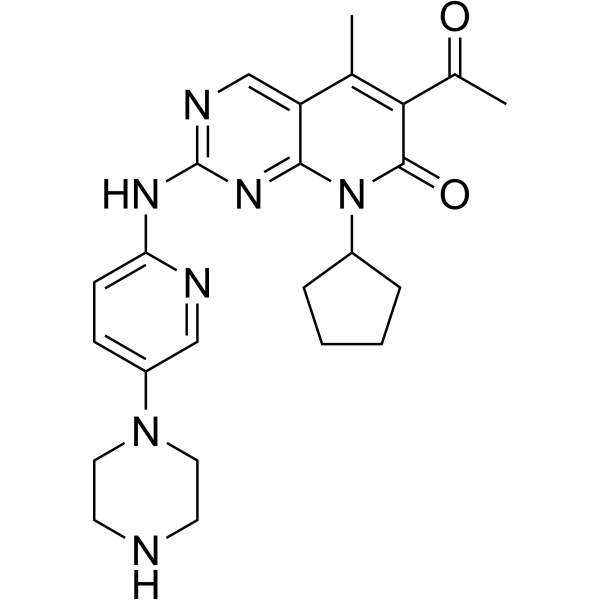 | [10] |
| Narirutin | 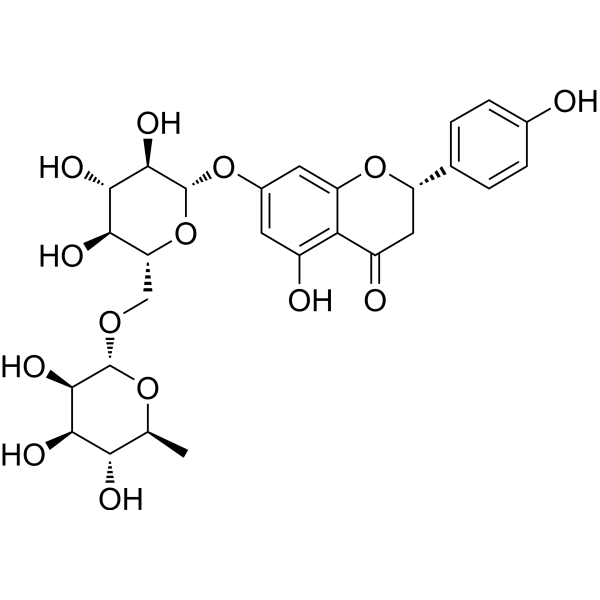 | [11] |
| Fenofibrate | 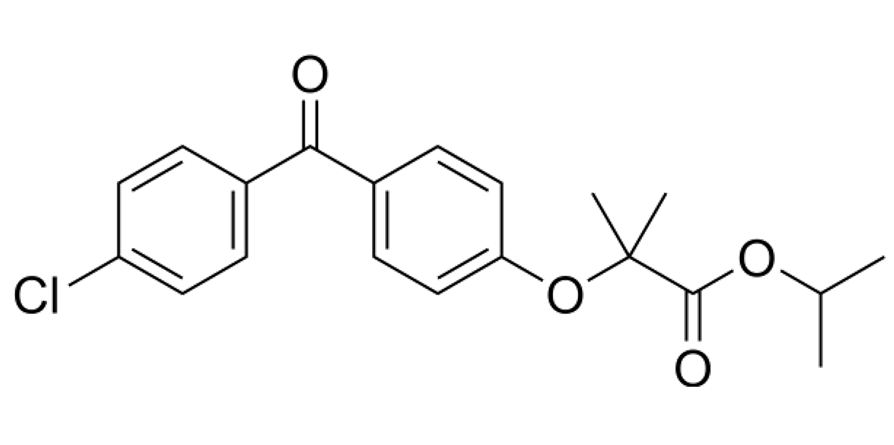 | [12] |
| Curcumin analog C1 | 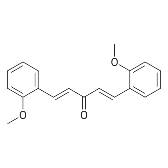 | [13] |

**Reference**

[1] D. Li, R. Shao, N. Wang, N. Zhou, K. Du, J. Shi, Y. Wang, Z. Zhao, X. Ye, X. Zhang, H. Xu, Sulforaphane Activates a lysosome-dependent transcriptional program to mitigate oxidative stress, Autophagy 17(4) (2021) 872-887.

[2] D. Kim, H.Y. Hwang, H.J. Kwon, A natural small molecule induces MAPT clearance via mTOR-independent autophagy, Biochem Biophys Res Commun 568 (2021) 30-36.

[3] C. Yang, Z. Zhu, B.C. Tong, A. Iyaswamy, W.J. Xie, Y. Zhu, S.G. Sreenivasmurthy, K. Senthilkumar, K.H. Cheung, J.X. Song, H.J. Zhang, M. Li, A stress response p38 MAP kinase inhibitor SB202190 promoted TFEB/TFE3-dependent autophagy and lysosomal biogenesis independent of p38, Redox Biol 32 (2020) 101445.

[4] Z. Niu, G. Tang, X. Wang, X. Yang, Y. Zhao, Y. Wang, Q. Liu, F. Zhang, Y. Zhao, X. Ding, X. Hao, Trigonochinene E promotes lysosomal biogenesis and enhances autophagy via TFEB/TFE3 in human degenerative NP cells against oxidative stress, Phytomedicine 112 (2023) 154720.

[5] M. Palmieri, R. Pal, H.R. Nelvagal, P. Lotfi, G.R. Stinnett, M.L. Seymour, A. Chaudhury, L. Bajaj, V.V. Bondar, L. Bremner, U. Saleem, D.Y. Tse, D. Sanagasetti, S.M. Wu, J.R. Neilson, F.A. Pereira, R.G. Pautler, G.G. Rodney, J.D. Cooper, M. Sardiello, mTORC1-independent TFEB activation via Akt inhibition promotes cellular clearance in neurodegenerative storage diseases, Nat Commun 8 (2017) 14338.

[6] P. Peng, D. Jia, L. Cao, W. Lu, X. Liu, C. Liang, Z. Pan, Z. Fang, Akebia saponin E, as a novel PIKfyve inhibitor, induces lysosome-associated cytoplasmic vacuolation to inhibit proliferation of hepatocellular carcinoma cells, J Ethnopharmacol 266 (2021) 113446.

[7] S.H. Kim, G. Kim, D.H. Han, M. Lee, I. Kim, B. Kim, K.H. Kim, Y.M. Song, J.E. Yoo, H.J. Wang, S.H. Bae, Y.H. Lee, B.W. Lee, E.S. Kang, B.S. Cha, M.S. Lee, Ezetimibe ameliorates steatohepatitis via AMP activated protein kinase-TFEB-mediated activation of autophagy and NLRP3 inflammasome inhibition, Autophagy 13(10) (2017) 1767-1781.

[8] V. Ammanathan, P. Mishra, A.K. Chavalmane, S. Muthusamy, V. Jadhav, C. Siddamadappa, R. Manjithaya, Restriction of intracellular Salmonella replication by restoring TFEB-mediated xenophagy, Autophagy 16(9) (2020) 1584-1597.

[9] Y. Li, M. Xu, X. Ding, C. Yan, Z. Song, L. Chen, X. Huang, X. Wang, Y. Jian, G. Tang, C. Tang, Y. Di, S. Mu, X. Liu, K. Liu, T. Li, Y. Wang, L. Miao, W. Guo, X. Hao, C. Yang, Protein kinase C controls lysosome biogenesis independently of mTORC1, Nat Cell Biol 18(10) (2016) 1065-77.

[10] Q. Yin, Y. Jian, M. Xu, X. Huang, N. Wang, Z. Liu, Q. Li, J. Li, H. Zhou, L. Xu, Y. Wang, C. Yang, CDK4/6 regulate lysosome biogenesis through TFEB/TFE3, J Cell Biol 219(8) (2020) e201911036.

[11] Z. Fang, Y. Xu, G. Liu, Q. Shao, X. Niu, W. Tai, T. Shen, M. Fan, M. Chen, L. Lei, W. Gao, Y. Song, Z. Wang, X. Du, X. Li, Narirutin activates TFEB (transcription factor EB) to protect against Acetaminophen-induced liver injury by targeting PPP3/calcineurin, Autophagy 19(8) (2023) 2240-2256.

[12] J. Yoo, I.K. Jeong, K.J. Ahn, H.Y. Chung, Y.C. Hwang, Fenofibrate, a PPARα agonist, reduces hepatic fat accumulation through the upregulation of TFEB-mediated lipophagy, Metabolism 120 (2021) 154798.

[13] J.X. Song, Y.R. Sun, I. Peluso, Y. Zeng, X. Yu, J.H. Lu, Z. Xu, M.Z. Wang, L.F. Liu, Y.Y. Huang, L.L. Chen, S.S. Durairajan, H.J. Zhang, B. Zhou, H.Q. Zhang, A. Lu, A. Ballabio, D.L. Medina, Z. Guo, M. Li, A novel curcumin analog binds to and activates TFEB in vitro and in vivo independent of MTOR inhibition, Autophagy 12(8) (2016) 1372-89.
