# Supplementary material for: Isoginkgetin protects against degeneration of ALS motor neurons via regulating the GSK-3β–TFEB signaling axis
Source: Pharmacol Res. 2026 May;227:108172. doi: 10.1016/j.phrs.2026.108172 (PMC13132972; doi:10.1016/j.phrs.2026.108172)
Supplement: Supplementary file 7 — Supplementary material [file mmc7.docx]

| Supplementary Table 6 Patient tissue samples from Netherlands Brain Bank | | | | | | | |
| --- | --- | --- | --- | --- | --- | --- | --- |
| Diagnosis code^1^ | | Autopsy^1^ | Sex^1^ | Age^1^ | Braak^1, 2^ | Amyloid^1^ | Region^1^ |
| Non-demented control | C1 | S18/112 | F | 95 | 3 | - | Middle frontal gyrus |
|  | C2 | S19/077 | F | 91 | 3 | - |  |
|  | C3 | S19/050 | M | 77 | 2 | - |  |
|  | C4 | S19/103 | M | 87 | 2 | - |  |
|  | C5 | S20/113 | M | 87 | 3 | - |  |
| ALS | A1 | S15/086 | F | 67 | - | - | Middle frontal gyrus |
|  | A2 | S16/073 | F | 53 | - | - |  |
|  | A3 | S16/088 | M | 57 | - | - |  |
|  | A4 | S18/052 | M | 78 | 2 | - |  |
|  | A5 | S12/111 | M | 62 | - | - |  |

^1^Provide from Netherlands Brain Bank

^2^Braak stage indicates
